# Uncovering the inertia of dislocation motion and negative mechanical response in crystals

**DOI:** 10.1038/s41598-017-18254-5

**Published:** 2018-01-09

**Authors:** Yizhe Tang

**Affiliations:** Shanghai Institute of Applied Mathematics and Mechanics, Shanghai University, Shanghai, 200072 China

## Abstract

Dislocations are linear defects in crystals and their motion controls crystals’ mechanical behavior. The dissipative nature of dislocation propagation is generally accepted although the specific mechanisms are still not fully understood. The inertia, which is undoubtedly the nature of motion for particles with mass, seems much less convincing for configuration propagation. We utilize atomistic simulations in conditions that minimize dissipative effects to enable uncovering of the hidden nature of dislocation motion, in three typical model metals Mg, Cu and Ta. We find that, with less/no dissipation, dislocation motion is under-damped and explicitly inertial at both low and high velocities. The inertia of dislocation motion is intrinsic, and more fundamental than the dissipative nature. The inertia originates from the kinetic energy imparted from strain energy and stored in the moving core. Peculiar negative mechanical response associated with the inertia is also discovered. These findings shed light on the fundamental nature of dislocation motion, reveal the underlying physics, and provide a new physical explanation for phenomena relevant to high-velocity dislocations.

## Introduction

Mechanical behavior of crystals is mainly determined by the ubiquitous linear defects, dislocations, thus dislocation behavior is the key to crystals’ mechanical performance. It’s been more than 80 years since the concept of dislocation was first proposed by Orawan^1^, Taylor^2^ and Polanyi^3^ independently in 1934. Since then, numerous endeavors have been made to understand the crystallographic, physical and mechanistic aspects of dislocations in various crystals^4^. As yet, the most fundamental nature of dislocation motion remains unjustified. The motion of dislocation converts strain energy irreversibly into thermal energy through radiation drag, phonon drag and electron drag processes^4,5^, thus is well accepted to be dissipative. On the other hand, inertial effects were also considered to be related to moving dislocations. The concept of dislocation inertia, appearing as “relativistic” or “dynamic” effect at first, was brought up to attention when theoretically studying a uniformly moving screw dislocation approaching to the transverse sound speed *c*_*t*_, with neglect of dissipation^6,7^. The displacement field in motion was found to be identical with that of a dislocation at rest^6,7^, apart from a “Lorentz contraction”. Consequentially, the time-varying displacement field yields a non-zero velocity field; and the kinetic energy was associated with dislocation velocity through the definition of effective dislocation mass, in analogy to classical mechanics^7^, and referred as “relativistic” or “dynamic” effect. Thereafter, the problem of a moving dislocation was extensively studied by Eshelby^8^, Weeterman^9,10^ and Markensskoff and Clifton^11,12^ among others, in the non-uniform motion case for both edge and screw dislocations. Gurrutxaga-Lerma *et al*.^13^ adopted the time-dependent elastic fields of moving dislocation, and developed a dynamic discrete dislocation plasticity model to study high strain rate deformations such as shock. An additional term was added to the dislocation mobility law to account for inertial effects at high velocities. Despite the fact that kinetic energy associated with moving dislocation has been long known, actual inertial motion of a dislocation, namely moving of a dislocation without driving force, has hitherto not been considered existing. In most real circumstances, dissipation processes dominate dislocation motion; whether inertial or not, the exhibited motion is always over-damped, making explicit identification of inertia almost impossible. The inertial nature of dislocation motion thus still remains hypothetical and lacks verification.

The mere supportive experimental evidences of inertia, though indirect, were reported when mechanically testing superconducting metals^14–16^. Plasticity enhancement was observed, with universality, when the metals were switching from normal to superconducting state^17,18^. The plasticity enhancement was generally believed to be attributed to the inertial overshooting of dislocations over obstacles when viscous drag suddenly becomes inactive in superconducting state^17,18^. However, it should be pointed out that, the inertia was deduced from the mechanical response, rather than being directly observed, and there also existed other interpretations that are not related to inertia, such as the changes in electron drag^15,19^, obstacle strength^20,21^ and mobile dislocation density^22^ when entering the superconducting state. Efforts have also been made by Indenbom and Estrin to distinguish between different dynamic models of plasticity enhancement^23^. These experimental results and different theoretical interpretations were summarized and reviewed by Kostorz^24^. So far there is still a dearth of experimental evidence demonstrating the existence of dislocation inertial motion. In recent years, atomistic simulations were also conducted in the case of dislocation-obstacle interactions^25,26^; however, no direct evidence of such motion was observed.

The present study utilizes atomistic model to quantify dislocation motion in conditions equivalent to that of a superconducting state in typical model crystals (hexagonal close-packed Mg, face-centered cubic Cu and body-centered cubic Ta), and explicitly demonstrates the occurrence of under-damped oscillatory motion of dislocation, which is a direct evidence of dislocation inertia, to reveal the hidden inertial nature of dislocation motion. In addition, a strikingly surprising phenomenon, negative mechanical response, which is generally impossible in natural materials, is discovered and explained in terms of inertial dislocation motion.

## Model and Method

The dislocations considered are edge and screw basal <*a*> dislocations in hcp Mg, 1/2 <1–10> (111) dislocations in fcc Cu and 1/2 <111> (2-1-1) dislocations in bcc Ta, respectively. Dislocations on the closest-packed (1–10) plane in Ta are also considered but not shown here for consistency, since screw 1/2 <111> (1–10) dislocations cross-slip to (2-1-1) plane^27,28^ while the edge ones cannot.

**Figure 1:**
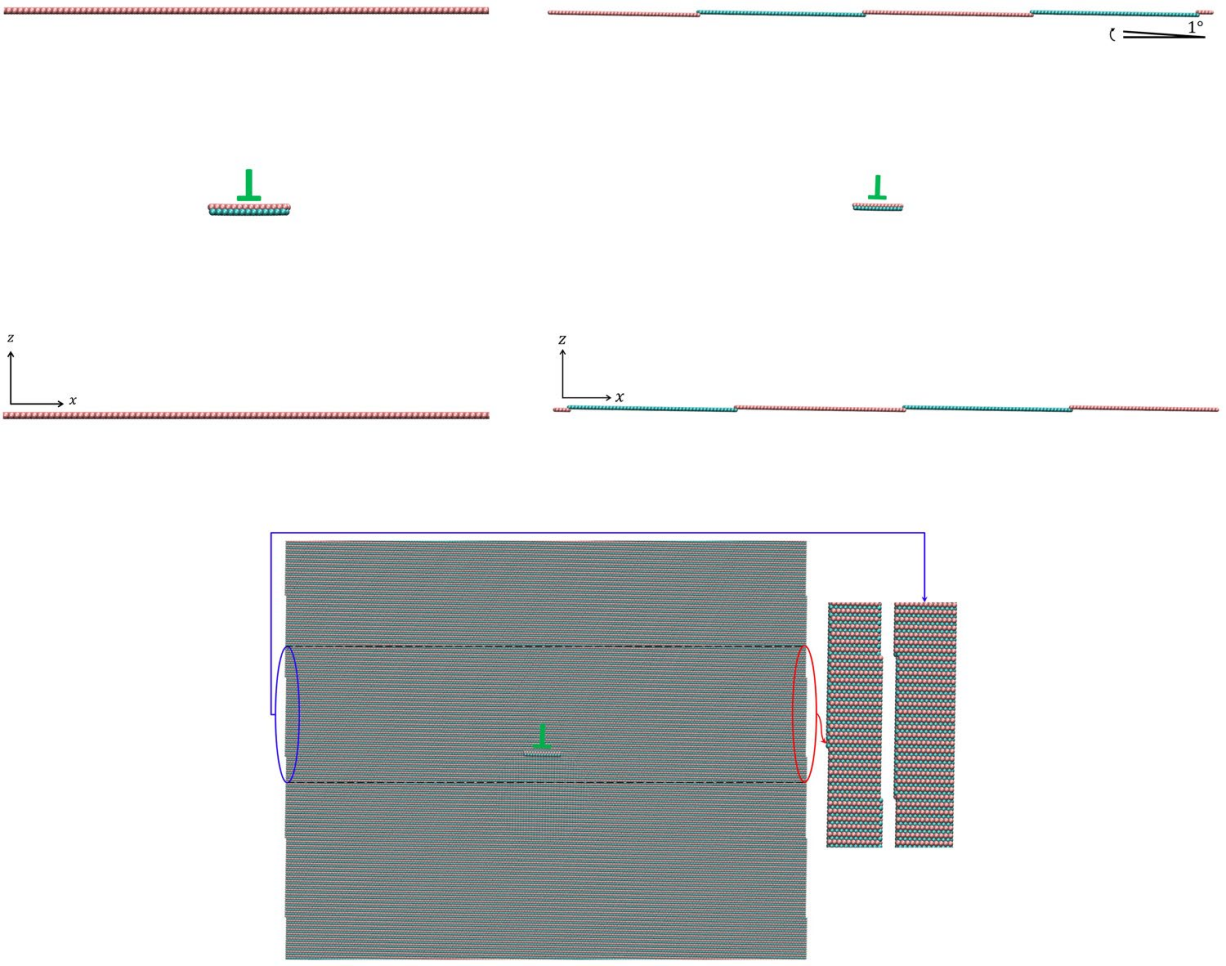
Schematic of the simulation cell for shear loading (upper left) and the inclined simulation cell for normal loading of an edge dislocation (upper right). The trimmed sample used is shown in the lower figures and seamless match between the left and right side is also illustrated.

Both shear and normal loading are considered. For shear loading, the crystal samples are rectangular with 100 nm length (x-direction, parallel to the dislocation glide direction) and 150 nm thickness (z-direction, perpendicular to glide plane). Along the width direction (y-direction), a unit length in the corresponding crystallography orientation is taken, and periodic boundary conditions (PBCs) are utilized to mimic infinite straight dislocations, as shown in Fig. 1. The dislocations are introduced by applying the corresponding anisotropic displacement filed of each dislocation^29^ to the intact crystal. For edge dislocations, PBCs are used in the dislocation glide direction directly after appropriately removing two layers of atoms in the *x* plane. For screw dislocations, shifted periodic boundary conditions^30,31^ are used. Surfaces are not present in the dislocation glide direction, and thus dislocations are able to move on the glide plane unlimitedly. These conditions actually mimic an infinite slab with an array of dislocations. The dislocation density, 7 × 10^9^ cm^−2^, somehow larger than that for annealed metals but smaller than deformed ones^32^, stays the same with that of a finite-long slab; whereas the distance a dislocation can move is infinite.

For normal loading of an inclined crystal containing an edge dislocation in Mg, the same sample mentioned above is rotated along the *y*-direction by 1°, and then is trimmed off to fit into a rectangular box. With carefully chosen sample length, the left side matches the right side seamlessly after dislocation introduction, and periodic boundary conditions are used in the glide direction, as shown in Fig. 1, to mimic an infinite slab. If the dislocation exists at the right side, it re-enters the sample from the left side at a position *n* layers below its original glide plane, where integer *n* satisfies *n* × *c* = *l*_*x*_ × tan 1° (*a* and *c* are the lattice constants of hcp Mg and *l*_*x*_ is the sample length). Actually, *n* is prescribed to be 3 first, then *l*_*x*_ is set to be 287 *a*, and the angle is thus determined to be 0.98°.

After dislocation introduction, energy minimization is performed. Loading is applied in a conventional manner. Six atomic layers at the bottom are fixed in the loading direction (*x*- and *y*-direction for edge and screw, respectively in shear, and *z*-direction in normal loading). Constant velocity in the loading direction is applied to six layers at the top to give constant strain rate; while forces are applied to give constant stress or stress rate. Along the other two directions, the bottom and top atoms are still free to move. For screw dislocation in Ta, shear is applied in the easy twinning direction, and the difficult anti-twining direction^27,28^ is not considered. In normal loading, the resultant normal stresses in the two lateral directions are relaxed by appropriately re-scaling the sample sizes, in order to maintain a uniaxial stress state. During loading, NVE ensemble is used, and the temperature rises from 0 K to at most 0.5 K at the end of the simulation for shear loading, and to at most 0.1 K for normal loading.

Shear loading at 77 K is also performed. The sample is heated up to 77 K and then kept at 77 K using NPT ensemble for 40 ps to achieve an equilibrium state. During loading, NVE ensemble is used again.

The potentials used are the EAM potential developed by Sun *et al*.^33^, and MEAM potential by Jim *et al*.^34^ and Wu *et al*.^35^ for Mg, EAM potential by Mishin *et al*.^36^ for Cu, and EAM potential by Ravelo *et al*.^37^ for Ta. For Mg, the potential is crucial for studies of non-basal dislocations, but less crucial for basal dislocations. Nevertheless, we use the EAM potential to gain a GPU acceleration^38,39^ of 30 times over the MEAM potential; meanwhile we always repeat relevant simulations using the MEAM potential to verify the consistency. Another EAM potential for Mg developed by Liu *et al*.^40^ was found to have better performance in predicting behavior of <*c*+ *a*> dislocations^41,42^; however, it predicted metastable screw basal dislocation^43^, and hence is not adopted here.

All molecular dynamics (MD) simulations in this study are performed using the LAMMPS code^44^. Common neighbor analysis (CNA)^45^ is used for defect (dislocations and surfaces) detection and VMD^46^ is used for visualization. Dislocation position tracking is processed automatically based on the CNA detection.

## Results

### Mechanics of dislocation motion

Crystals, when subject to external loading, deform elastically first until the stress reaches a critical level, the Peierls stress, and then dislocations start to move. The motion of dislocations hereafter accommodates the elastic deformation and reduces the stress in turn. The stress state and the deformation in this problem, known as dislocation mechanics, are governed by three equations.

The first is geometry equation that simply relates dislocation motion to plastic deformation, regardless of stress states. This is known as the Taylor-Orowan Equation first given by Taylor^2^ in 1934 and then a strain-rate form by Orowan in 1940^47^:

1 $${\gamma }_{p}=\rho bl$$

where *γ*_*p*_ is the plastic shear strain, *ρ* the dislocation density, and *b* the magnitude of dislocation Burgers vector. In Taylor’s original version^2^, *l* is the total length along the slip plane. For the case of a dislocation moving an arbitrary distance, the equation is

1′ $${\gamma }_{p}=\rho b{\rm{\Delta }}l$$

where Δ*l* is the displacement of a dislocation. A detailed derivation of Eqs (1) and (1′) based on volume average is given in the Supplementary material.

The second equation relates dislocation motion to the stress change ${\rm{\Delta }}\tau$ caused by the motion. Since dislocation motion generates a certain amount of strain that doesn’t need stress to maintain (plastic strain *γ*_*p*_), the stress corresponding to such amount of strain is thus elastically released:

2 $${\rm{\Delta }}\tau =-G{\gamma }_{p}$$

where *G* is the shear modulus. Accordingly, the total stress *τ* only arises from the remained elastic strain:

2′ $$\tau =G{\gamma }_{e}=G({\gamma }_{t}-{\gamma }_{p})$$

where *γ*_*e*_ is the elastic shear strain and *γ*_*t*_ the total shear strain applied. Eq. (2′′) can be considered as a “dynamic” equilibrium equation, and clearly the mechanical response *τ* depends on the competition between the applied and released strains *γ*_*t*_ and *γ*_*p*_.

The third equation relates dislocation motion to the stress *τ* that causes the motion, which is the constitutive equation. If the motion is dissipative only, it takes the form^4^

3 $$b\tau =Bv\quad (\tau \ge {\tau }_{c})$$

where *B* is the viscous drag coefficient consisted of contributions from phonon radiation *B*_*r*_, phonon drag *B*_*p*_ and electron drag *B*_*e*_^5^, and *v* is dislocation velocity and *τ*_*c*_ is the Peierls stress. Apparently, dissipative motion requires shear stress (mechanical work) to maintain.

If the motion is considered to be inertial only, it takes the form

3′ $$M\frac{dv}{dt}=b(\tau -{\tau }_{c})$$

where *M* is the effective mass of a dislocation in motion^7,13,48,49^.

If the motion, in a general case, is both inertial and dissipative, it takes the form^48^

3′′ $$M\frac{dv}{dt}+Bv=b(\tau -{\tau }_{c})$$

Substituting Eqs (1′) into (2′) yields:

4 $$\tau =G({\gamma }_{t}-\rho b{\int }_{0}^{t}vdt)$$

**Figure 2:**
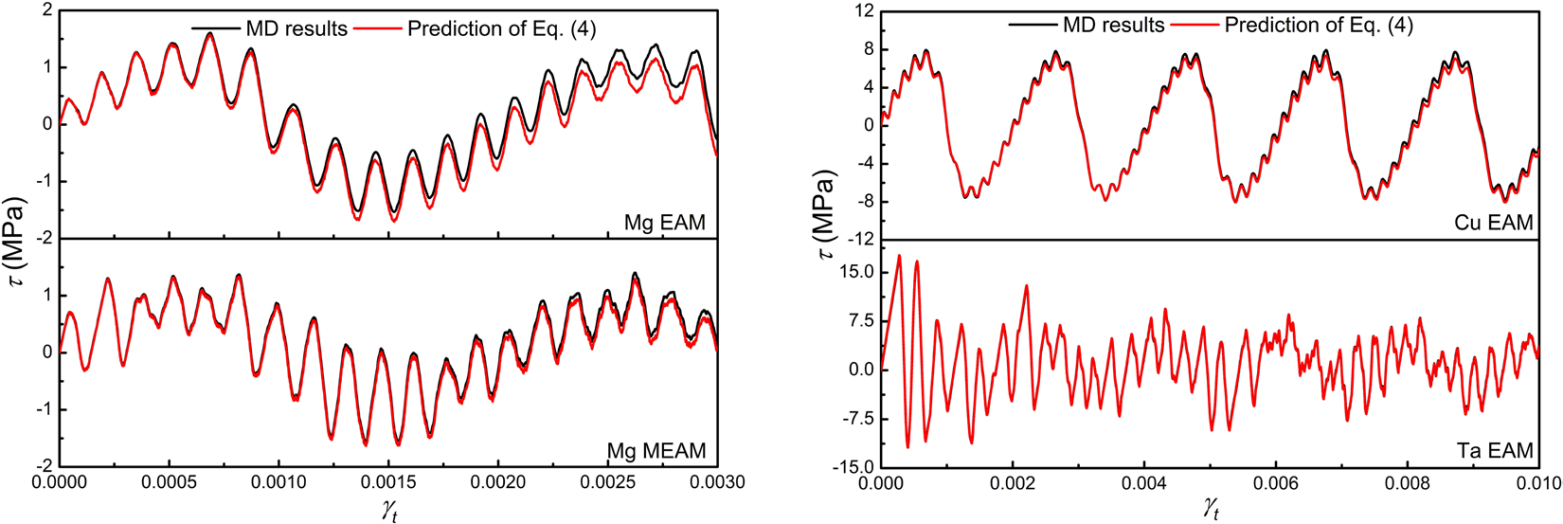
Comparison of the stress ~ strain (*τ* ~ *γ*_*t*_) responses of Mg, Cu and Ta crystals containing a single edge dislocation sheared at a strain rate of 10^6^ s^−1^ predicted by Eq. (4) (red line) with that calculated from MD simulations (black line). Reasonable agreement is observed.

In the governing equation (4), *G* and *b* are material parameters, *ρ* is determined by microstructure state, and *γ*_*t*_ is determined by external loading condition. Apparently, the constitutive relationship *v* = *v* (*τ*) serves as a kernel function and once determined, dislocation motion and stress evolution can all be solved. Using the constitutive relationship extracted from atomistic simulations, the validity of Eq. (4) is quantitatively examined first by comparison with atomistic simulations of an edge dislocation in simple shear, as shown in Fig. 2. Given the fact that thermal effects were included in atomistic simulations but not in Eq. (4), the agreement could be considered as reasonable.

### Inertia of dislocation motion in shear loading at constant strain rates

Dislocations, when moving in crystals, are always subjected to dragging forces. Various dragging mechanisms were analyzed and evaluated by Alshits and Indenbom^50^, and the main causes of dislocation dragging in pure systems were found to be the interactions with electrons and phonons. Radiation of elastic waves and thermal phonons is also important^4,5^. In the present study, atomistic simulations were intentionally performed at very low temperature *T* ~ 0 to 0.5 K, such that phonon drag *B*_*p*_ from phonon-dislocation interaction is minimized. There is also no electron drag *B*_*e*_ in the empirical potentials (EAM^51,52^ and MEAM^53^). Phonon radiation *B*_*r*_ from the moving core thus becomes the only remaining dissipative process, mimicking the conditions for dislocation motion in superconducting state^14–16^.

The Peierls stresses for all the edge and screw dislocations studied here, determined at a relatively low strain rate of 10^5^ *s* ^−1^, are listed in Table 1.

**Table 1:** Peierls stress (in MPa) for the edge and screw dislocations in Mg, Cu and Ta.

	Mg (EAM)	Mg (MEAM)	Cu	Ta
edge	0.3	0.75	1.3	16
screw	3.3	50	15.5	130

**Figure 3:**
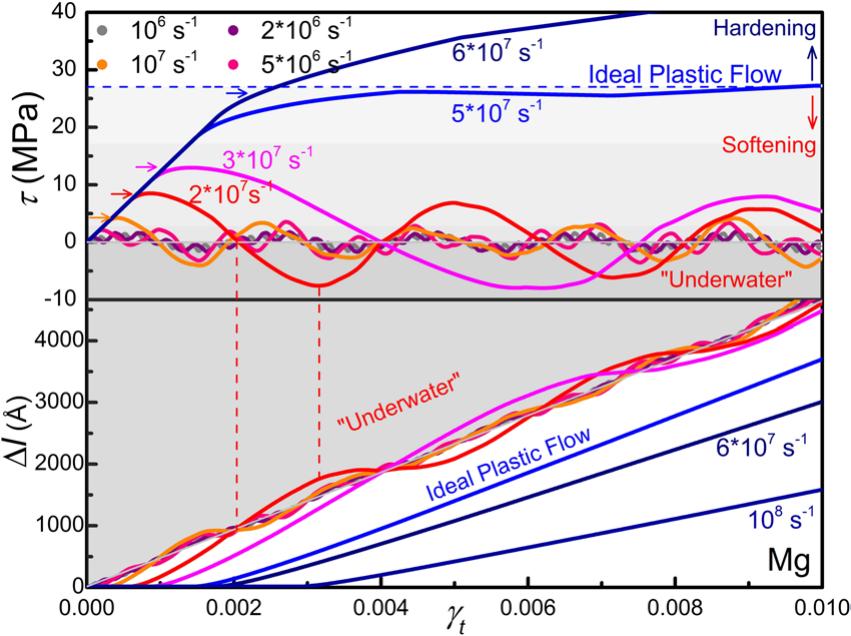
Stress-strain (*τ* ~ *γ*_*t*_) response and dislocation displacement-strain (Δ*l* ~ *γ*_*t*_) curve for Mg crystal containing a single edge dislocation sheared at various strain rates. Yielding is indicated by arrows. Softening region is light-greyed and negative stress region (“Under Water”) is dark-greyed. Shear stress oscillates around the zero-stress axis in the softening region due to inertia. In Δ*l* ~ *γ*_*t*_ plot, dislocation overshooting the equilibrium position (diagonal) due to dislocation inertia is evident. The overshot region (“Under Water”) is also dark-greyed.

**Figure 4:**
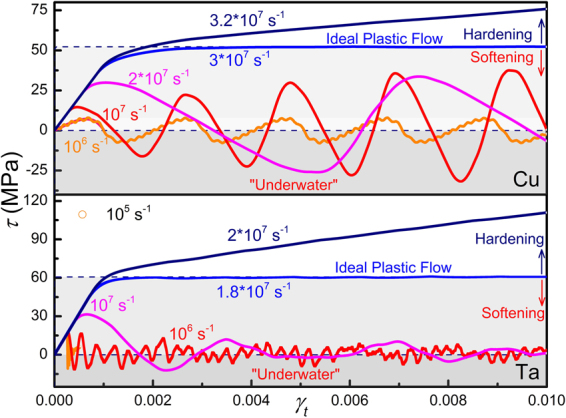
Stress-strain (*τ* ~ *γ*_*t*_) response for Cu and Ta crystals containing a single edge dislocation sheared at various strain rates. Softening region is light-greyed and negative stress region (“Under Water”) is dark-greyed.

Figures 3 and 4 show the stress-strain (*τ* ~ *γ*_*t*_) response of different metals containing a single edge dislocation loaded at various shear strain rates (10^3^ *s* ^−1^ to 10^9^ *s* ^−1^). The shear stress *τ* is obtained by averaging over the whole sample. Two distinct features are noticeable, the rate effect and *negative mechanical response*. In Fig. 3 it’s clearly seen that the yield stress, indicated by arrows, increases with strain rate. The rate effect is generally believed to be associated with dissipation processes, but here can be interpreted simply in terms of the competition between the applied strain rate *dγ*_*t*_/*dt* and releasing rate of strain *dγ*_*p*_/*dt* in Eq. (2′): when *dγ*_*t*_/*dt* is low enough that *dγ*_*p*_/*dt* caused by dislocation motion can always catch up, there will be no stress build-up and yielding (maximum *τ* where *dτ*/*dt* = 0) occurs at a low stress level that close to the Peierls stress; as *dγ*_*t*_/*dt* increases, dislocation motion falls behind at first and stress builds up, but the dislocation would keep accelerating until *dγ*_*p*_/*dt* reaches *dγ*_*t*_/*dt* at a higher stress level and then yielding occurs. There exists a critical strain rate at which the stress stays constant (*dτ*/*dt* ≡ 0 or *dγ*_*t*_/*dt* ≡ *dγ*_*p*_/*dt*) after yielding, exhibiting ideal plastic flow. Above this critical strain rate (*dγ*_*t*_/*dt* > *dγ*_*p*_/*dt*), *γ*_*p*_ is not able to catch up with *γ*_*t*_ anymore, thus the stress *τ* continues to increase, exhibiting hardening behavior. This is unavoidable since dislocation velocity usually has an upper-bound. The maximum velocity a dislocation can reach at a certain stress level, determined by the constitutive relation, can also be a bound (*v* reaches only 1019 m/s at stresses up to 120 MPa for Ta). Up to a strain rate of 10^9^ *s* ^−1^, the dislocations studied here are all found to be moving in the subsonic regime. When materials deform at rates that exceed the capability of dislocation motion, stress will continue to increase and other deformation modes, twinning, phase change or fracture, will take place instead.

Below the critical strain rate, there are occasions that *dγ*_*p*_/*dt* caused by dislocation motion catches up with *dγ*_*p*_/*dt* (*dγ*_*t*_/*dt* < *dγ*_*p*_/*dt*), and thus the stress *τ* decreases with increasing strain *γ*_*t*_, exhibiting softening behavior. Similar results are also observed for Cu and Ta (Fig. 4). The critical strain rate observed here, 10^7^ s^−1^, seems too high. Noting that the dislocation density adopted is too high, 7 × 10^9^ cm^−2^. If a typical value in annealed metals^32^, 10^7^ × cm^−2^, is adopted, the critical strain rate would drop to 10^4^ s^−1^, according to Eq. (4). If phonon drag, pinning and dislocation-obstacle interactions are also present, dislocation would move more slowly and the critical strain rate is expected to further drop to a more realistic level.

The other distinct feature, observed in the softening region, is more significant and surprising. For example, in Fig. 3, after reaching a maximum, the stress τ continues to decrease until reaching a minimum with almost the same amplitude of that for the maximum in the negative stress zone (*τ* < 0, referred as “Underwater” hereafter), and then increases again. This is very surprising, remembering that the crystal keeps being strained at all times. The occurrence of *negative mechanical response* (*τ* < 0 while *γ* > 0) in simple and ordinary crystals is very unusual, provided that it is not a dynamic effect of stress waves. If dislocation motion is over-damped as usual, the plastic strain *γ*_*p*_ caused by dislocation motion would never exceed *γ*_*t*_, and thus the stress *τ* would never, according to Eq. (2′), go below the “Water Level” (*τ* = 0), not to mention the “Underwater” (*τ* < 0) zone. Whereas the results for all the three metals clearly show that the stress τ indeed oscillates around the “Water Level” periodically, exhibiting typical under-damped oscillatory feature even when the dislocation velocity here is somehow low (maximum dislocation velocity is 140 m/s for Mg at 10^6^/s, about 5% of *c*_*t*_).

If we turn to dislocation behavior, the under-damped oscillatory motion is more explicit. The dislocation displacement Δ*l* extracted from atomistic simulations is also shown in Fig. 3 for Mg (see Cu and Ta results in the Supplementary material). The diagonal represents the instantaneous equilibrium position at which dislocation motion releases the applied strain exactly, namely *γ*_*p*_ ≡ *γ*_*t*_ or *τ* ≡ 0, and the upper-right half (greyed area) represents the “Underwater” zone. It’s clearly seen that (see the red curve for example), the dislocation accelerates with *γ*_*t*_, and gains a maximum velocity (seen from the slope) instead of stopping when crossing the equilibrium position, a typical inertial overshooting phenomenon. As the dislocation continues advancing into the “Underwater” zone, it gets slowed down by the negative stress, reaching a stationary state when *τ* reaches a minimum, and re-accelerates again when the stress turns positive.

Given the surprisingness of the oscillations observed for common dislocations subject to conventional loading, the possible dynamic effects of stress waves were carefully examined.

Firstly, it’s easily noted that in constant strain rate loading, the average stress *τ* exactly equals to *Gγ*_*t*_, provided that kinetic energy is negligible. If a shear wave is present and has traveled a fraction *β* of the sample thickness *l*_*z*_ and the rest stays un-sheared, the average stress *τ* takes the volume average over the sheared (*γ* = *γ*_*t*_/*β*) and un-sheared (*γ* = 0) parts, *G* [*γ*_*t*_/*β* • *βl*_*z*_ + 0 • (1−*β*) *l*_*z*_]/*l*_*z*_ = *Gγ*_*t*_. Thus, elastic waves alone wouldn’t result in any stress oscillation, and deviation from the linearity can only arise from dislocation motion.

**Figure 5:**
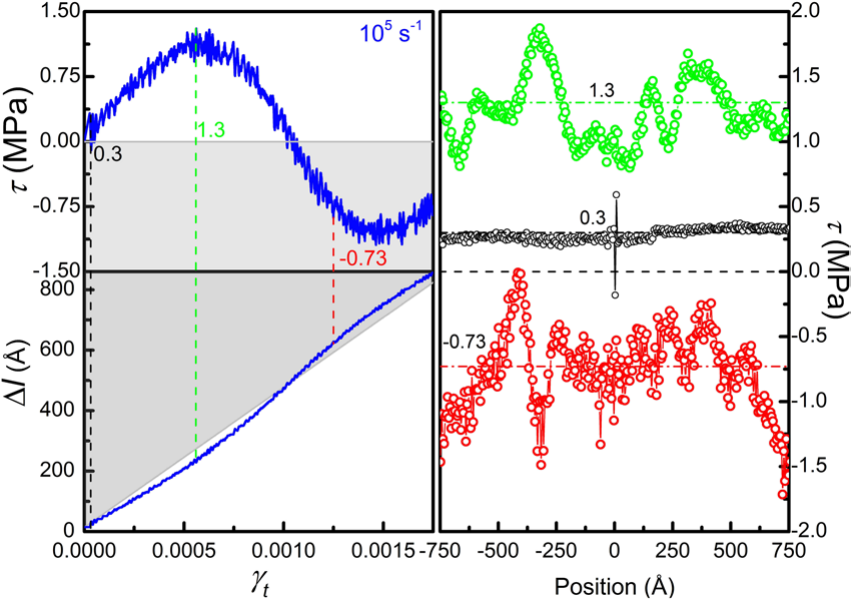
Stress-strain (*τ* ~ *γ*_*t*_) and dislocation displacement-strain (Δ*l* ~ *γ*_*t*_) curve for an edge dislocation in Mg at 10^−5^ s^−1^ (left); profiles of shear stress along the thickness direction at different stress levels (right).

Secondly, at relatively low strain rate, the stress waves would reach an equilibrium state before dislocation starts moving. Figure 5 shows the stress *τ* and dislocation displacement Δ*l* at a strain rate of 10^5^ *s* ^-1^ for an edge dislocation in Mg. Oscillations are observed for both the stress and dislocation motion. The shear stress profiles along the thickness direction (from −75 to 75 nm, and dislocation resides at the very center) is also shown. Each data point represents an average over a slab of two atomic layers. It is seen that, at a shear stress level of 0.3 MPa, right before the dislocation starts moving, the stress is quite homogeneous and no obvious waves are present. At a maximum stress of 1.3 MPa, the stress profile becomes inhomogeneous and elastic waves do appear. These waves are not caused by loading, but a result of unloading caused by dislocation motion. At a negative stress of -0.73 MPa, although elastic waves are still present, all stresses are negative, and no local positive stresses are found, noting that at the moment the dislocation still holds a positive velocity, as shown in the Δ*l* curve in Fig. 5. This observation actually excludes the possibility that the positive dislocation velocity arises from a local dynamic positive stress when the average stress is negative, and validates that dislocation motion is indeed inertial. The *negative mechanical response* (*τ* < 0 while *γ* > 0) is thus a result of dislocation inertia.

**Figure 6:**
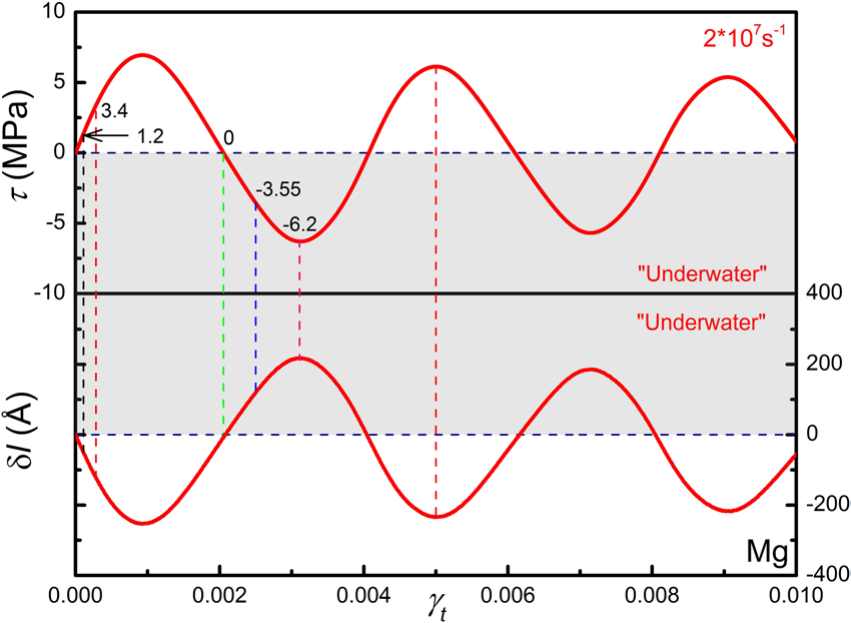
Stress ~ strain (*τ* ~ *γ*_*t*_) response and relative dislocation displacement-strain (δ*l* ~ *γ*_*t*_) curve for edge dislocation in Mg homogeneously sheared at 2 × 10^7^ s^−1^. The negative stress and overshot regions (“Under Water”) are dark-greyed. The two oscillations differ in phase by π.

**Figure 7:**
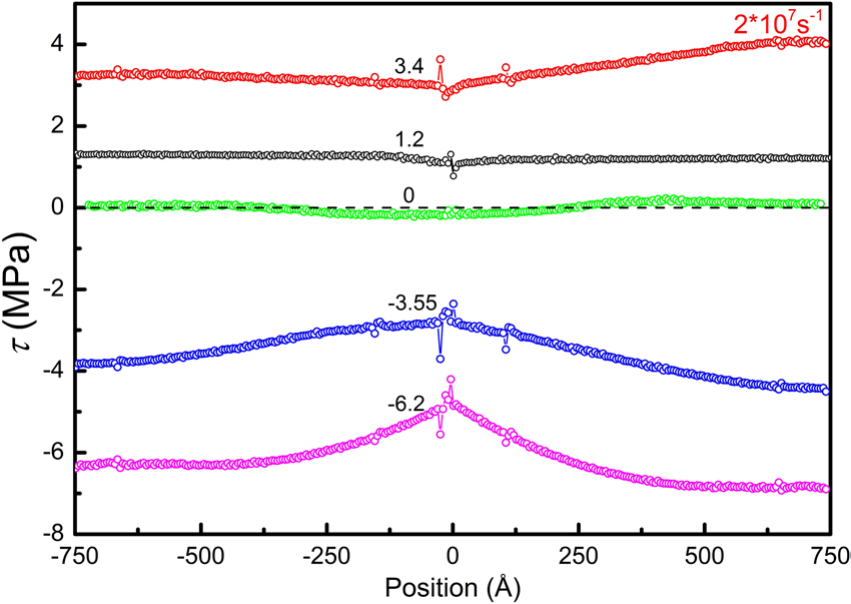
Profiles of the shear stress along the thickness direction at different stress levels for an edge dislocation in Mg homogeneously sheared at 2 × 10^7^ s^−1^.

Thirdly, with increasing strain rates, elastic waves would be more significant. In order to evaluate the results obtained at high strain rates, an alternative loading method is adopted to reduce strong elastic waves. A homogeneous shear strain is applied to the sample by displacing all atoms accordingly, and the amount of strain varies linearly with time, which also yields a constant strain rate with no elastic waves. Figure 6 shows the stress *τ* and relative dislocation displacement *δl* at a strain rate of 2 × 10^7^ *s* ^−1^ for an edge dislocation in Mg. The relative dislocation displacement *δl* = Δ*l − γ*_*t*_ */ρb*, is calculated by taking the instantaneous equilibrium position of dislocation, *γ*_*t*_ */ρb*, rather than its initial position, as a reference state, to better show the oscillation. The stress-strain response is nearly the same with that shown in Fig. 3 (red curve). Figure 7 shows the shear stress profiles along the thickness direction. It is seen that, at a shear stress of 1.2 MPa, the dislocation already moved but the stress-strain curve stays linear, a typical rate effect or over-shot phenomenon. At a shear stress of 3.4 MPa, dislocation motion leads to unloading wave travelling outwards, exhibiting a V-shape stress profile. When the average stress decreases to zero, the dislocation achieves its maximum velocity, as seen in Fig. 6. Again, at a negative stress of -3.55 MPa, all stresses are negative and the dislocation still holds a positive velocity. As the dislocation slows down, loading waves surpass the unloading wave generated by dislocation motion, and the stress profile becomes inverted V-shape. It is noted that, if the oscillation is a dynamic effect of stress waves, then the oscillations of shear stress *τ* and dislocation velocity *v* should be in phase. As shown in Fig. 7, a clear difference in phase by π between *τ* and *δl* is seen, and thus a difference in phase by π/2 between *τ* and *v* is expected, in analogy to that of a one-dimensional harmonic oscillator. Hence it is confirmed that the under-damped oscillatory motion of dislocation observed here is of inertia nature, not a resultant oscillation of stress waves, and dislocation motion is indeed dominated by inertia in the adopted conditions.

### Inertia of dislocation motion in combined shear loading conditions

At higher velocities, the inertia of dislocation motion is expected to be more significant. The reason that it’s not seen at high strain rates in Figs 3 and 4 is because the crystals are over-driven and thus the oscillatory feature disappears. In order to visualize the inertia of fast-moving dislocations, we turn to different loading conditions.

**Figure 8:**
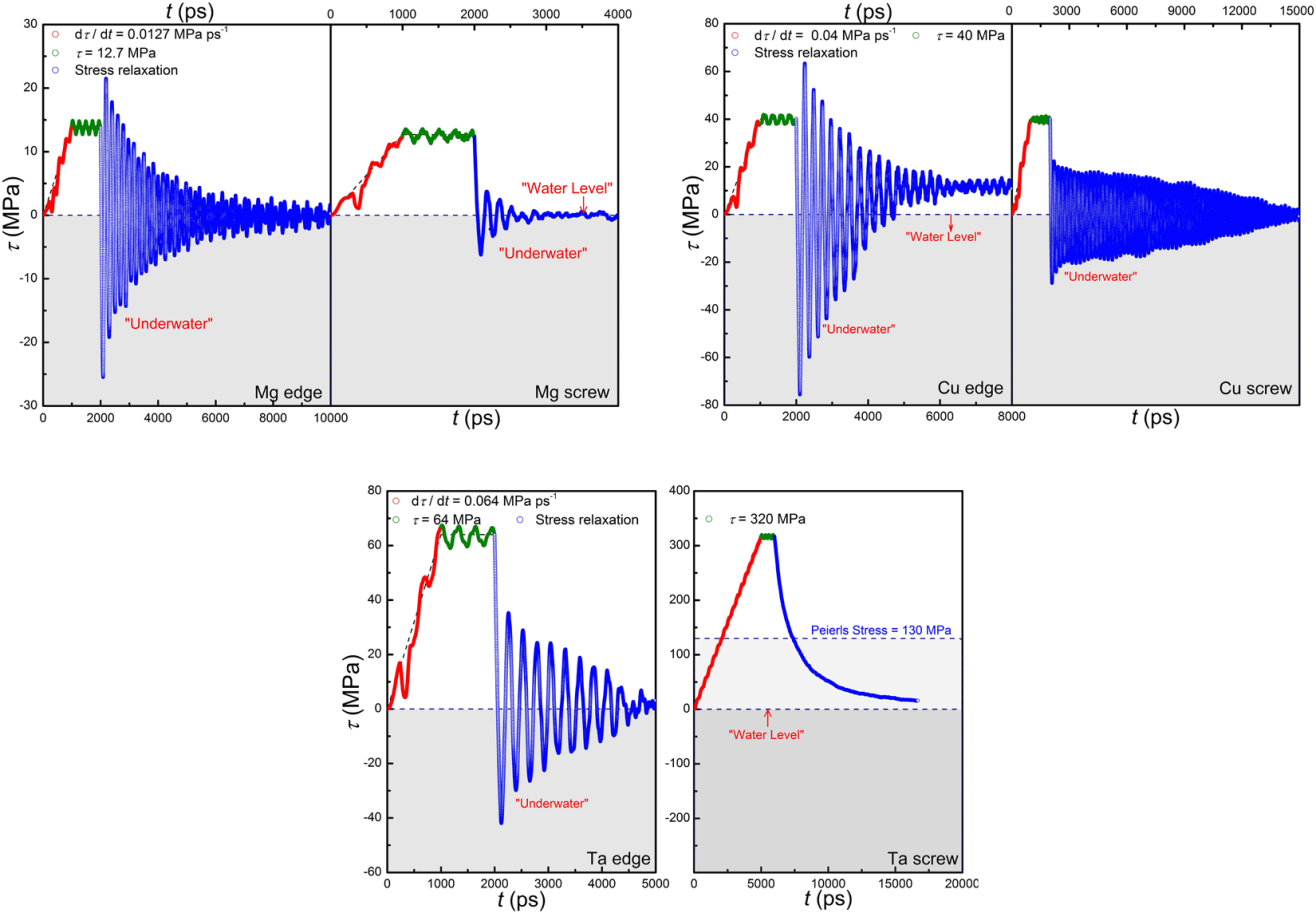
Stress evolution (*τ* ~ *t*) for Mg, Cu and Ta crystals containing an edge or a screw dislocation in a combined loading condition of constant stress rate (10^−6^ *G* ps^−1^), constant stress (10^−3^ *G*) and stress relaxation. Negative stress region (“Under Water”) is dark-greyed. In all cases except for screw dislocation in Ta, shear stress oscillates around the zero-stress axis at a frequency of gigahertz during stress relaxation due to inertia, and gets fully relaxed in about 10 nanoseconds.

A shear stress increasing at a constant rate, instead of a shear strain, is applied first for 1,000 ps until it reaches *G*/1000, then the stress is kept constant for another 1,000 ps (creep condition) to allow the dislocation to acquire a uniform velocity. The shear strain is then fixed to let the system evolve conservatively (stress-relaxation condition). Figure 8 shows the stress evolution for both edge and screw dislocations in Mg, Cu and Ta. It is seen that, during stress relaxation, the shear stress didn’t monotonously drop to zero, but oscillated around the “Water Level” until its magnitude decayed to zero, exhibiting significant under-damped oscillatory behavior again.

**Figure 9:**
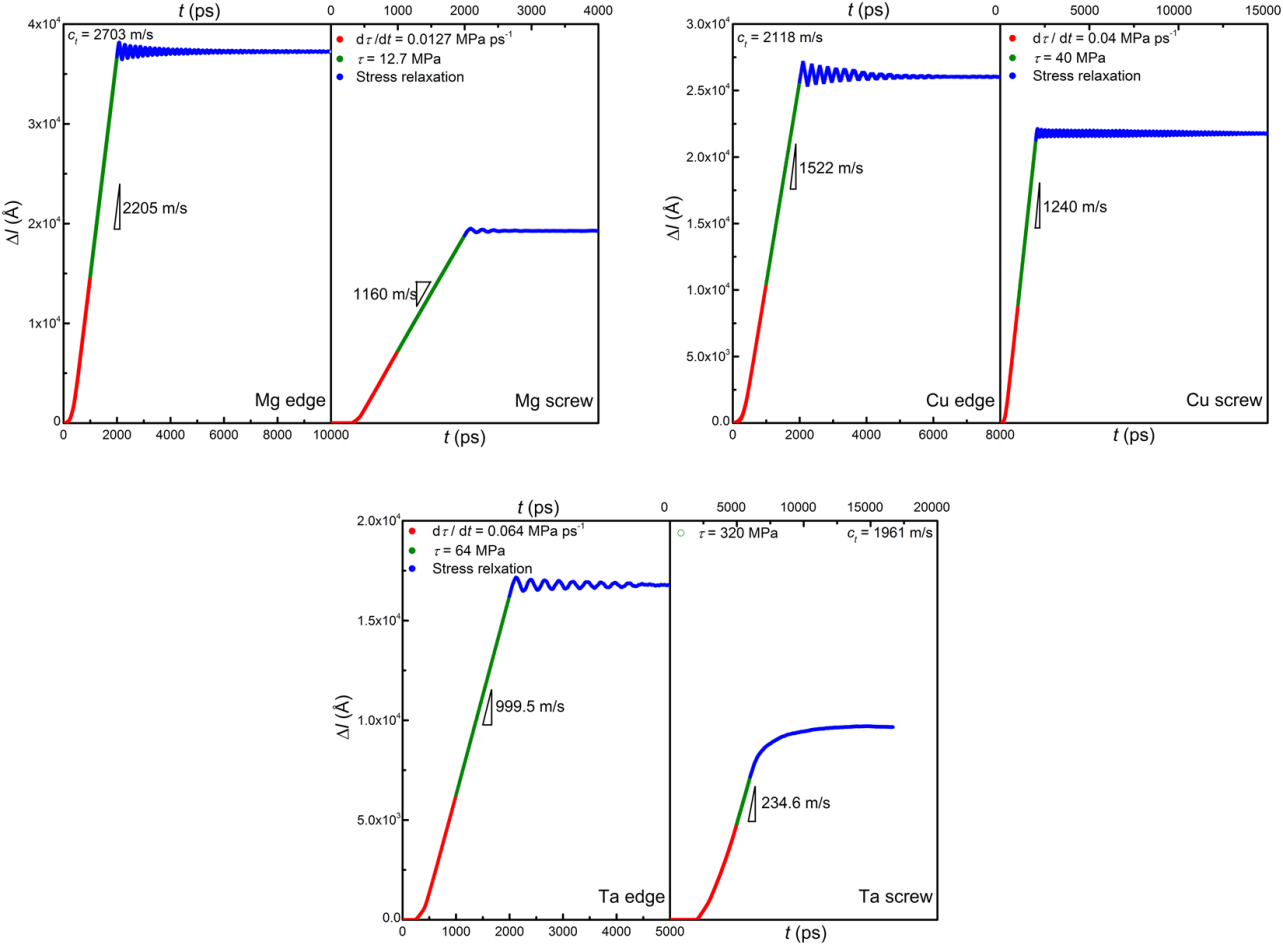
Dislocation displacement ~ time (Δ*l* ~ *t*) curves for Mg, Cu and Ta crystals containing an edge or a screw dislocation in a combined loading condition mentioned above.

The relaxation time is typically around 10 nanoseconds, and the stress gets fully relaxed to a very low level; while in the experiments of superconducting metals, the largest stress relaxation reported was only about 53%^19^. The oscillation frequency falls in the range of 3~6 GHz, an extremely high level. The extracted dislocation motion, as shown in Fig. 9, also shows clear under-damped oscillatory behavior and demonstrates that at high velocities dislocation motion is still dominated by inertia.

In all cases except for the screw dislocation in Ta, the dislocation oscillates around its equilibrium position at a frequency of gigahertz during stress relaxation and eventually stops in about 10 nanoseconds. The oscillations of τ and Δ*l* differ in phase by π, namely τ reaches a minimum when Δ*l* reaches a maximum. In the only exceptional case of the screw dislocation in Ta, the dislocation has a compact core and thus high Peierls stress (~130 MPa) and low mobility (only 200 m/s at 320 MPa). This is very typical in body-centered cubic metals^54,55^. It is observed that, the dislocation velocity decreases monotonously and slowly to zero (see Fig. 9) without oscillating, a typical over-damped feature. Correspondingly, the shear stress drops monotonously to a stress level of 16 MPa without oscillating. The over-damped dislocation motion should be attributed to the low dislocation velocity (~200 m/s) and high energy dissipative rate associated with the motion of the compact core. Dislocation inertia, although not dominant in this case, is still evident. It is noted that the dislocation continues to move at stresses even below the Peierls stress, 130 MPa (see light-grayed area in Fig. 8), indicating that the inertial effect is non-negligible even at low velocities.

**Figure 10:**
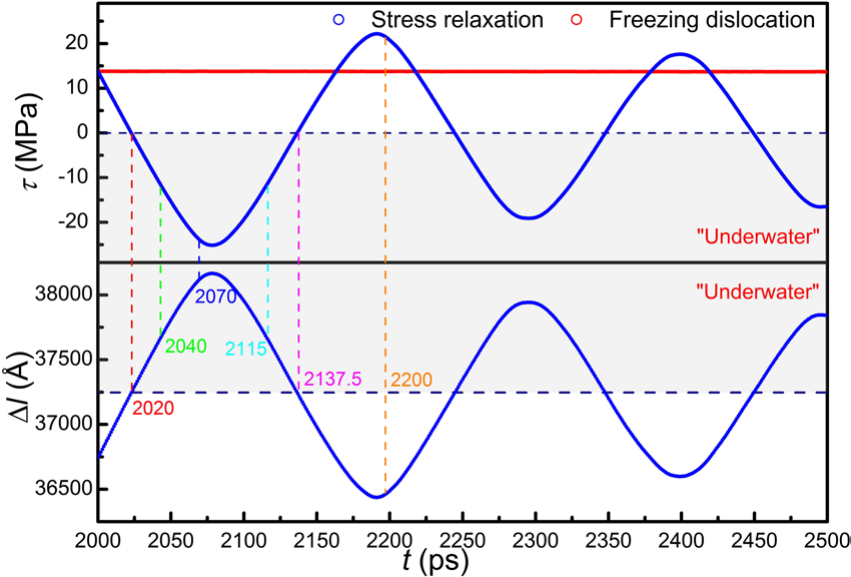
Stress evolution (*τ* ~ *t*) and dislocation displacement ~ time (Δ*l* ~ *t*) curve for edge dislocation in Mg during stress relaxation. A linear velocity field has been deducted before stress relaxation. Stress is found to be constant when dislocation is frozen.

**Figure 11:**
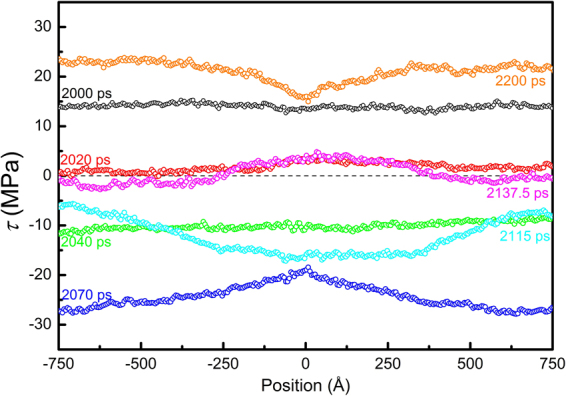
Profiles of the shear stress along the thickness direction at different stress levels during stress relaxation of an edge dislocation in Mg.

Similarly, stress waves might also be present during stress-relaxation simulations. When dislocation moves at a uniform velocity, the system undergoes a constant-rate plastic flow. The velocity field associated with the plastic flow would cause strong waves if the sample boundaries are fixed suddenly. In order to reduce the undesired waves, a linear velocity field corresponding to the plastic flow is deducted from the full velocity field after the dislocation reaches a uniform velocity (at 2000 ps), and the boundaries are then fixed to perform stress-relaxation simulations. Figure 10 shows the evolution of stress and dislocation motion for an edge dislocation in Mg, and similar results are obtained for both the stress and dislocation motion. A clear difference in phase by π between *τ* and Δ*l* is seen. For comparison, the dislocation is also frozen by fixing atoms in a 5 × 1 nm block containing the dislocation. It is seen that, without dislocation motion, the stress stays constant. Thus, it is validated that the stress oscillation is indeed a result of dislocation motion, rather than elastic waves. The stress profiles at different time steps are shown in Fig. 11. At 2000 ps, the stress profile is quite homogeneous. Once again, at 2040 ps, the average stress is negative, −10 MPa, and the stress profile is homogeneous; whereas the dislocation still holds a positive velocity. V-shape and inverted V-shape profiles are also observed, resulting from the interaction of unloading wave generated by dislocation motion with sample boundaries.

Sample-size and potential effects were also considered for Mg, and consistent results were obtained (see details in the Supplementary material). It is thus concluded that, dislocation motion in crystals is indeed of inertial nature, and the inertial nature is even more fundamental than dissipation. The hidden inertial effect is visible only when dissipative processes become insignificant at conditions similar to that utilized in the present study (see edge and screw dislocations in Mg and Cu, and edge dislocation in Ta); when dissipative processes dominate, the inertial effect still exists but just becomes less visible (screw in Ta) or invisible.

### Origin of the inertia of dislocation motion

Dislocation motion, even in under-damped mode, still requires energy to overcome the energy barrier (known as Peierls-Nabarro barrier) and compensate energy dissipation. In the case of stress relaxation simulations, there is no mechanical work provided, and thus the energy required to achieve inertial motion at negative stress levels can only come from internal and must be of kinetic nature.

**Figure 12:**
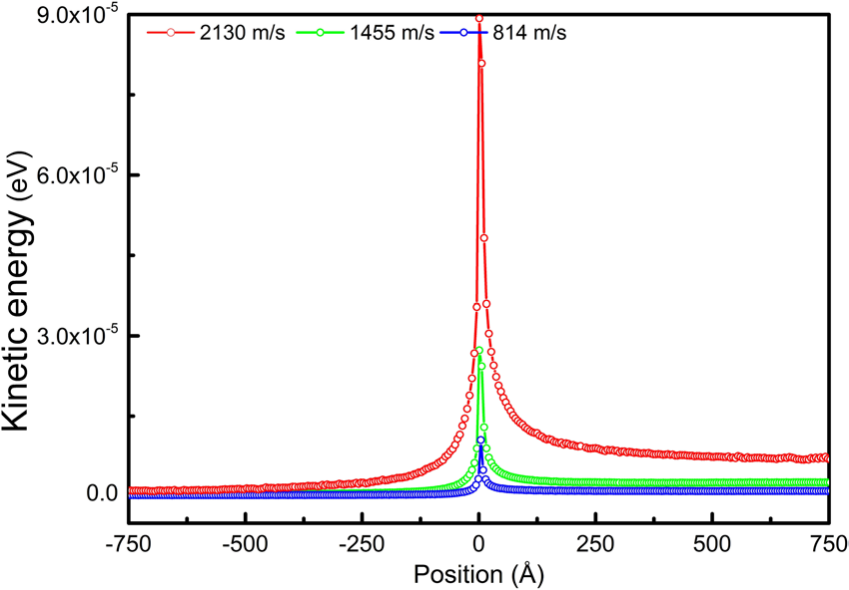
Profiles of the average kinetic energy along the thickness direction at different dislocation velocities (edge dislocation in Mg).

The profiles of kinetic energy along the thickness direction for an edge dislocation in Mg moving at different velocities are shown in Fig. 12. Since the dislocation is continuously moving on the glide plane, the average volume is taken as a slab of two atomic layers rather than a cylinder. It is evident that, dislocation glide plane has the highest kinetic energy, and majority of the kinetic energy is stored in the vicinity of the glide plane.

**Figure 13:**
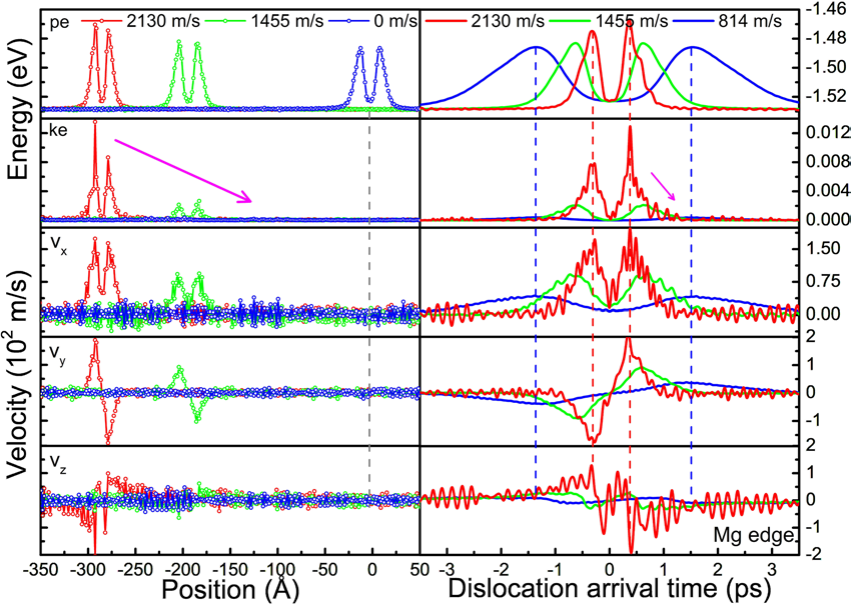
Spatial distributions (left) and temporal evolution (right) of the potential energy *pe*, kinetic energy *ke* and particle velocities *Vx*, *Vy* and *Vz* of the atoms in the glide plane of an edge dislocation at different velocities in Mg. For temporal evolution, only one representative atom is chosen since all atoms in the glide plane are identical. Considerable kinetic energy is carried in the moving dislocation core.

The spatial distributions of the potential, kinetic energy and particle velocities of atoms in the glide plane are also shown in Fig. 13 (left). The two peaks in potential energy profile indicate the positions of the two partial cores. It is seen that, dislocations at rest and in motion have insignificant differences in potential energy but significant difference in kinetic energy and particle velocities. The highest potential energy in the dislocation core is ~0.04 eV above the cohesive energy of Mg, −1.53 eV. At a dislocation velocity of 2130 m/s, the highest kinetic energy in the core is ~0.01 eV; while far away from the core in the glide plane, the kinetic energy is only 10^−5^ to 10^−4^ eV; and far away from the glide plane, the kinetic energy is only 10^−6^ to 10^−5^ eV. The dislocation in motion indeed carries considerable kinetic energies in the moving core, and the kinetic energy and particle velocities are highly correlated with dislocation velocity *v*. It is also noted that, the particle velocity profiles exhibit a clear pattern, not disordered, indicating that the kinetic energy carried by the dislocation is of mechanical type, not thermal. As the dislocation moves, the ordered velocity pattern mechanically propagates with the moving dislocation. When the velocity *v* slows down to zero, the stored kinetic energy and particle velocities in the core all vanish to the background level accordingly. Similar results were also observed for screw dislocation in Mg, and both edge and screw dislocations in Cu and Ta (see the details in the Supplementary material).

The temporal evolution of the potential, kinetic energy and particle velocities of a representative atom in the dislocation glide plane is also shown in Fig. 13 (right). The two pulses in the potential energy indicate the passage of the two individual partial cores. As the dislocation passes by, the atom is kinetically activated and experiences two kinetic energy pulses. So the motion of dislocation indeed converts part of the strain energy into kinetic energy, which will be stored in the core and mechanically propagating with the dislocation. This energy conversion process during dislocation motion actually gives a definite physical picture of the origin of dislocation inertia. As long as the energy conversion process is happening, there will be inertia and the inertia is independent of dissipation/radiation processes. In other words, dislocation motion is intrinsically inertial, no matter later on the kinetic energy gets dissipated/radiated rapidly or not.

When studying transonic and supersonic dislocations, Gumbsch and Gao^56^ and Jin *et al*.^57^ pointed out that the energy radiation (energy loss) from a moving dislocation determines how difficulty a dislocation can go from subsonic to transonic, and to supersonic. When approaching the sound speed *c*_*t*_, the radiation increases significantly, making transition from subsonic to transonic extremely difficult. Besides, as shown by Frank^7^ for a screw dislocation and by Gurrutxaga-Lerma *et al*.^13^ for an edge dislocation based on continuum elasticity theory, the elastic energy of a dislocation also increases dramatically as its velocity approaches *c*_*t*_, preventing the dislocation from surmounting the sound barrier. However, dislocations can indeed go transonic or supersonic if they are created as supersonic at high stress concentration and are driven by high stresses. In Gumbsch and Gao and Jin *et al*.’s considerations, the energy radiation is evaluated by calculating the kinetic energy generated by dislocation moving over a unit area^56,57^ in atomistic simulations. The total kinetic energy is generally believed to be consisted of contributions mainly from emission of elastic waves and thermal phonons. Here we clearly demonstrate that, in addition to the well-known radiated elastic waves and thermal phonons, there is also considerable kinetic energy of mechanical nature stored in the moving core of a dislocation. Since dislocation velocities here are still in the subsonic regime, the emission of elastic waves is not expected to be significant.

**Figure 14:**
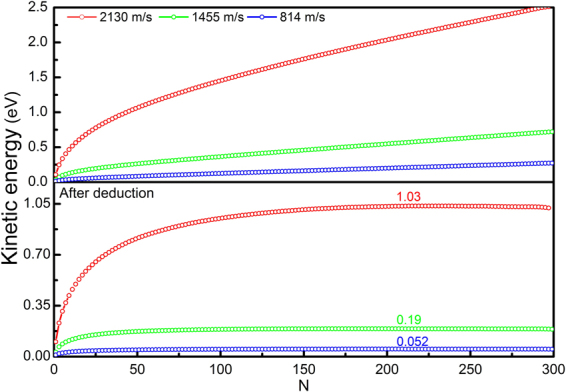
The total kinetic energy as a function of slab size *N* (upper). The energy after deduction of a homogeneous kinetic energy distribution is shown in the lower plot.

As proposed by Frank^7^, Kocks^48^ and Hirth *et al*.^49^, a simple and convenient physical quantity can be used to measure dislocation inertia, the effective dislocation mass *M*, which correlates the stored energy in a dislocation to its velocity *v*. A rigorous form of *M* that can be used in the equation of motion of a dislocation has been proposed by Hirth *et al*.^49^ and Gurrutxaga-Lerma *et al*.^13^, using the total energy’s derivative with respect to dislocation velocity *v*. The specific expression, however, depends on the solutions of the displacement and velocity fields of a moving dislocation. Considering the importance of *M* as a measure of dislocation inertia, we attempt to evaluate the effective dislocation mass *M* from the atomistic simulations. Since the dislocation considered here is moving uniformly and the derivative is inaccessible, as an alternative, *M* is taken as 2/*v* ^2^ times the kinetic energy, in analogy to classical mechanics (also see Hirth *et al*.^49^). The kinetic energy associated with a moving dislocation can be easily evaluated in elasticity theory; however, in atomistic simulations, ambiguousness arises. There will always be contributions from thermal vibrations and even possible elastic waves in the total kinetic energy. Velocity field corresponding to constant-rate plastic flow of materials when dislocation motion reaches a steady state also contributes to the total kinetic energy. As a result, accurately evaluating the kinetic energy associated with a moving dislocation in atomistic simulations becomes difficult. In Fig. 14, the total kinetic energy of a slab containing an edge dislocation at the very center in Mg is plotted as a function of *N*, where 2 *N* is the total number of atomic layers in the slab. Apparently, the total kinetic energy scales with the slab thickness. In the vicinity of dislocation glide plane (small *N*), the kinetic energy increases rapidly. As *N* increases, the kinetic energy becomes almost linear, indicating a homogeneous kinetic energy distribution in the far field. As a first approximation, this homogeneous kinetic energy is deducted from the total kinetic energy to account for thermal energies and other contributions, as shown in the lower plot in Fig. 14. It is seen that, after deduction, the kinetic energy reaches a plateau as *N* increases, and the plateau energy is defined as the kinetic energy stored in a moving dislocation. It is found that, at a dislocation velocity of ~800, 1500 and 2100 m/s, the kinetic energy is ~0.05, 0.2 and 1 eV, respectively. The effective mass *M* of the dislocation with √3*a* length, is 0.6, 0.7 and 1.8 *m*_0_, respectively, where *a* is the lattice constant of Mg and *m*_0_ the mass of Mg atom. It turns out that the effective dislocation mass *M* is neither a constant nor a linear function of dislocation velocity *v*; as *v* increases, *M* increases dramatically.

In Frank’s elasticity consideration^7^, the kinetic energy of a moving dislocation was only a result of the time-varying displacement field; the cause of the kinetic energy was indefinite there. It is speculated that^4^, acquisition of kinetic energy might happen when atoms in the dislocation core drop from the top of the Peierls-Nabarro potential barrier. Whereas in the present study, it is clearly shown that, this is not happening, at least from the perspective of an individual atom. It is seen from the temporal evolution of the potential and kinetic energy in Fig. 13 that, their variations are strictly in phase, and when the potential energy starts dropping after reaching a maximum (top of the barrier), the kinetic energy drops as well instead of acquiring extra boost. In fact, both the potential energy and the kinetic energy stored in the core are propagating with the dislocation, not interconverting conservatively. This actually gives us a clue that, the specific cause of the kinetic energy stored in a moving dislocation core might arise from outside of the core. It may perhaps be related to the radiation reaction that arises when a moving dislocation continually interacts with its own emitted wave field^8,58^. Further effort should be made towards fully understanding of the specific cause of the kinetic energy in the core.

When travelling at a velocity of 2130 m/s (~80% of *c*_*t*_), the maximum particle velocity in the dislocation core is ~ 200 m/s; whereas the mean square root (MSR) velocity at a temperature of 0.5 K is much smaller, only 13 m/s. As a result, there will be only weak interaction (exchange of quasimomentum) between the fast-moving particle in the core and the thermal phonons. The phonons thus stay inactive and exert no drag *B*_*p*_ on dislocation motion. Besides, after the atoms in the core get activated, there is no obvious velocity standing or tailing and the velocity vanishes to background level rapidly, as shown in Fig. 13. Hence there won’t be many new phonons emitted and the phonon radiation from the core *B*_*r*_ is also negligible. Elastic wave emission might exist but is not expected to be significant in the subsonic regime. That probably explains why dislocation motion is under-damped and the hidden inertia is visible in this condition. When moving at lower velocity, the particle velocity in the core decreases to the MSR velocity level, and interaction between the two velocities increases, leading to increased phonon drag *B*_*r*_. In addition, longer staying time (dislocation width over *v*) also allows more interacting time and enhanced viscous drag and hence less obvious under-damped motion or even over-damped motion (see Ta screw in Fig. 8). Another extremity is that, when dislocation velocity *v* approaches to the transverse sound speed *c*_*t*_, the energy radiation increases dramatically, as shown by Gao *et al*.^56^ and Jin *et al*.^57^. Although inertia still exists, dislocation motion is expected to be dominated by damping again, even at low temperature and without electron drag.

It is also worth noting that, as seen in the spatial distribution of the potential energy in Fig. 13, the width of the dislocation core indeed contracts “relativistically”, as predicted by Frank^7^. The contraction is ~30% when the dislocation moves at 2130 m/s (35% of the longitudinal sound speed *c*_*l*_), much larger than that predicted by Frank^7^, ~7% for an edge dislocation. The significant contraction was also considered to be one of the reasons that a subsonic dislocation can hardly go transonic in real systems, since transonic dislocations have much wider cores^56,57^.

### Tensile response in compression due to inertia

Dislocation motion is driven by shear stress, not normal stress, and can release only shear strain. In a more general case, when a normal stress *σ* is applied to a plane that has an inclination angle *α* to the dislocation glide plane, the dislocation would be driven by the resolved shear stress *τ*_*r*_ = *σ* cos*α* sin*α*, where cos*α* sin*α* is the Schmid factor, and thus plastically releases the normal strain by

1′′ $${\varepsilon }_{p}=\,\cos \,\alpha \,\sin \,\alpha \cdot \rho b{\rm{\Delta }}l$$

Accordingly, the normal stress *σ* can be expressed as

2′′ $$\sigma =E{\varepsilon }_{e}=E({\varepsilon }_{t}-{\varepsilon }_{p})$$

where *E* is the elastic modulus, *ε*_*e*_ the elastic normal strain and *ε*_*t*_ the total normal strain applied. Similarly, if *ε*_*p*_ exceeds *ε*_*t*_ due to dislocation motion, negative normal stress *σ* can also be achieved.

**Figure 15:**
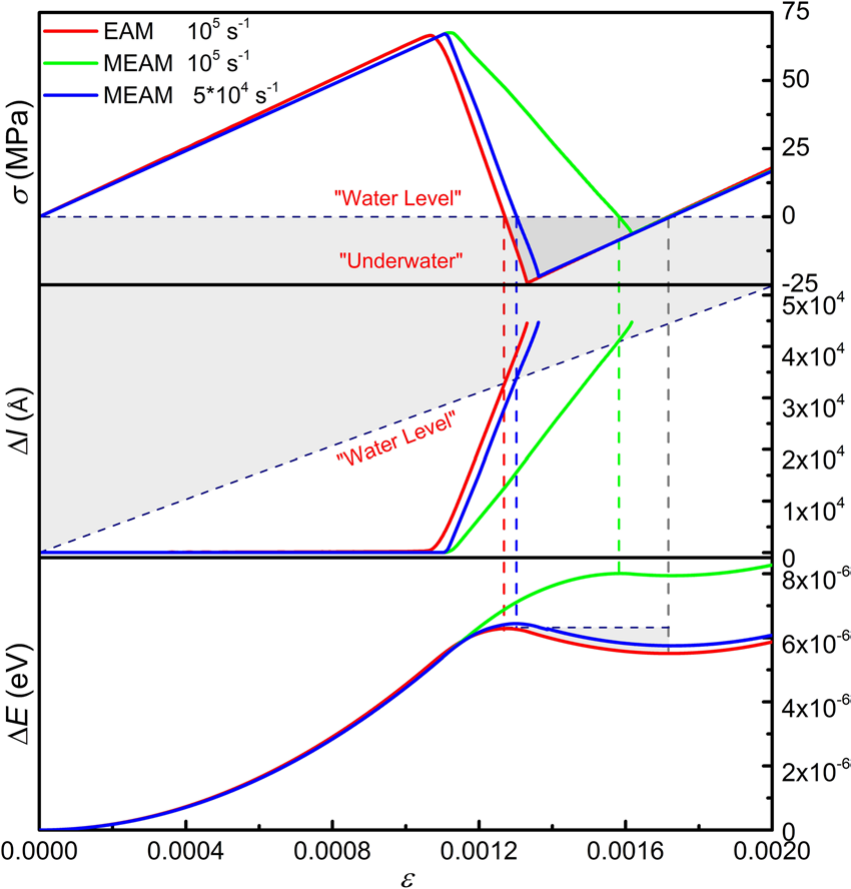
Normal stress *σ*, dislocation displacement Δ*l* and energy gain per atom Δ*E* as a function of normal strain *ε* for an inclined Mg crystal containing an edge dislocation loaded at constant normal strain rates of 10^5^ and 5 × 10^−4^ s^−1^. The normal of the dislocation glide plane deviates from the loading axis by 1°. Negative normal stress region (“Under Water”) is dark-greyed and negative normal stress is observed using both EAM and MEAM potentials.

Here we show an example of *negative mechanical response* in normal loading, namely tensile stress in compression. A periodic inclined sample is created by a novel trimming method. A small inclination angle of 1 degree is adopted, and both the dislocation line direction and sample lateral direction are set periodic. The variations of normal stress *σ*, dislocation displacement Δ*l* and energy gain per atom Δ*E* are shown in Fig. 15. It’s seen that, the stress *σ* deviates from linear elasticity after the onset of the dislocation. After the dislocation acquires a high velocity, the stress *σ* starts decreasing rapidly, due to rapid releasing of normal strain *ε* by dislocation motion. At a certain amount of strain, the stress *σ* indeed goes negative and continues decreasing until the dislocation eventually gets absorbed by the lower surface. After absorption, the stress increases linearly again. The energy gain reaches a maximum when the stress crosses the “Water Level” (*σ* = 0), and then decreases due to the negative mechanical work (*ε* > 0, *σ* < 0). When the stress becomes positive again, the energy gain also increases again.

Simulations using the MEAM potential^34,35^ also show similar results. Since the Peierls stress predicted by the MEAM is higher than that for the EAM, 0.75 *vs*. 0.3 MPa, and the dislocation velocity is only half of that for the EAM (see the slopes in Δ*l ~ ε* curves in Fig. 15), the negative response is of less significance in this case. However, if half of the strain rate is adopted, strain release caused by dislocation motion becomes overwhelming (*dε*_*p*_ */dt* $\gg$ *dε*_*t*_ */dt*), and the negative response is significant again.

**Figure 16:**
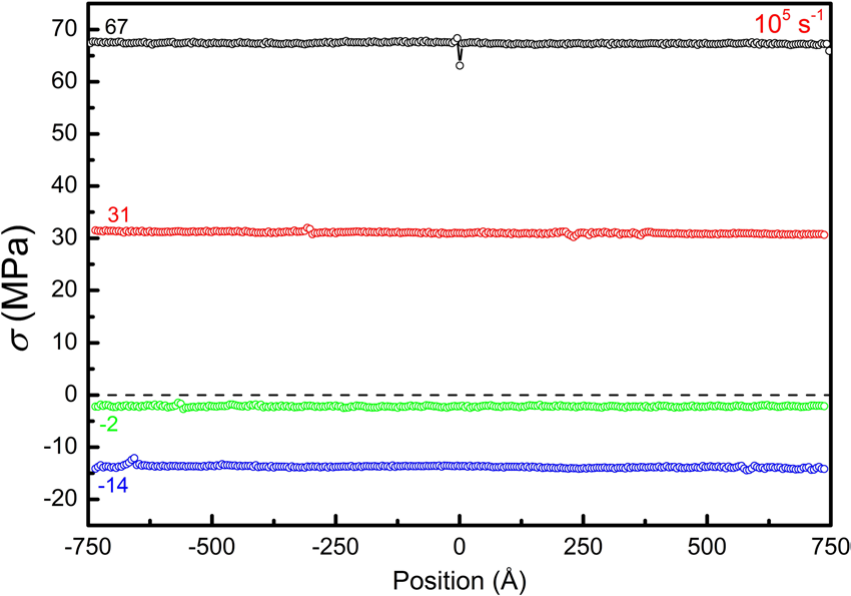
Profiles of the normal stress along the thickness direction at different stress levels during normal loading of edge dislocation in inclined Mg sample (EAM potential, 10^5^ s^−1^).

The stress profiles are also examined in normal loading. Figure 16 shows the profiles of normal stress *σ* in Mg with the EAM potential, loaded at a strain rate of 10^5^ s^−1^. The stress profiles at different stress levels are found to be quite homogeneous. At negative stress levels of −2 and −14 MPa, no local positive stress is observed. Since the stress state is uniaxial, the resolved shear stress is also negative and the dislocation indeed holds positive velocities at these negative stresses.

It is noticed that, in all the three cases, when the stress *σ* crosses the “Water Level”, the dislocation displacement Δ*l* also happens to cross the diagonal, suggesting that the ‘dynamic’ equilibrium equation Eq. (2″) for inclined normal loading is also accurate. After dislocation absorption, the stress *σ* becomes positive again at the same strain in all the three cases. This observation is not a coincidence. For a given sample, the maximum distance a dislocation can travel is geometrically determined. Thus in Eq. (2″), the applied strain *ε*_*t*_ required to satisfy *σ* = 0 is also determined, regardless of the strain rates and potentials adopted.

In structural elements, negative-stiffness does exist (buckled column and pre-stressed springs) and is always connected to instabilities^59^. Elastic composites containing negative-stiffness phase can even be stable^60^. In solids, phase change can lead to negative-stiffness^59^ too. Similarly, plasticity and fracture in solids can also be considered as instabilities, and negative-stiffness (e.g. decreasing loads with increasing displacement) indeed appears. However, in the present study, in addition to the “negative-modulus” (decreasing stress with increasing strain), the stress itself also goes negative. The occurrence of *negative mechanical response* (*τ* < 0 while *γ* > 0) should be viewed as a “dynamic” effect, resulting from the inertial dislocation motion, and the dislocation can be considered as an emerging “mass” connected to the elastic media.

## Discussion and Conclusions

It is concluded that, via atomistic simulations, under-damped inertial motion of dislocation does exist in the absence of phonon drag and electron drag, and the nature of dislocation motion is both inertial and dissipative. The inertia originates from the kinetic energy imparted from strain energy and stored in the core during motion. The simulations are performed at low temperature, in order to mimic the super-conducting state. This condition allows for uncovering of explicit under-damped oscillatory motion of dislocations at both low and high velocities. The only dissipative process present here is the phonon radiation from dislocation core. At higher temperatures, rapid increase in phonon density results in enhanced phonon drag; hence inertia effect becomes less significant (see the results for an edge dislocation in Mg, Cu and Ta at T = 77 K in the Supplementary material). Besides, effects of electrons are also present in states other than supper-conducting state, making the inertia effects even less significant in most real circumstances.

Nevertheless, the demonstrated inertia phenomenon and its underlying physics revealed here are still of great generality and significance. When dislocation moves at high velocities, such as in high rate deformation and shocks^4,13,49^, inertial effects, whether explicitly visible or not, have to be taken into consideration. Treating dislocations as quasi-static might introduce unphysical artifacts in these conditions^13^. The kinetic energy associated with dislocation motion might make dislocation activities, such as cross-slip, dislocation-obstacle interactions and so on, energetically more active. New physical understanding of the relevant phenomena (work-hardening, dynamic recovery, fatigue and so on) and even novel phenomena might arise. It is recently reported that^61^, a dislocation moving at a high velocity towards a free surface, got bounced back surprisingly, instead of being absorbed. In the perspective of dislocation inertia, this phenomenon is inevitable: the surface can absorb the configuration (potential) energy, but can only rebound (not absorb) the high particle velocities of the incident dislocation, which in turn re-creates a dislocation carrying the same amount of kinetic energy traveling in the opposite direction. The energetically unfavorable dislocation reaction during reflection, *b* → −*b* + 2*b* was realized with the assistance of the emerging high kinetic energy at high dislocation velocity. Under some circumstances, motion of high-velocity dislocations was reported to transition to rough and twinning modes and produce debris and twin embryo^62^. The kinetic energy carried by the dislocation might also play an important role in these processes.

Finally, as inspired by the discovery of the negative mechanical response demonstrated in Fig. 15, dislocation inertia can also be used as a new means to achieve novel mechanical responses through manipulation of dislocation behavior.

## Electronic supplementary material

Supplementary Material
