# Supplementary material for: Uncovering the inertia of dislocation motion and negative mechanical response in crystals
Source: Sci Rep. 2018 Jan 9;8:140. doi: 10.1038/s41598-017-18254-5 (PMC5760717; doi:10.1038/s41598-017-18254-5)
Supplement: Supplementary file 1 — Supplementary Material [file 41598_2017_18254_MOESM1_ESM.pdf]

Supplementary Material for

"Uncovering the inertia of dislocation motion and negative mechanical response in crystals"

Yizhe Tang[1,*]

[1]Shanghai Institute of Applied Mathematics and Mechanics, Shanghai University, Shanghai 200072, China

[*]Email: yzhe.tang@gmail.com

This file includes:

Derivation of Taylor-Orowan Equation

Cu and Ta in constant rate shear: Dislocation displacement-strain curve

Sample size effects: Mg

Potential effects: Mg

Energies of dislocation at rest and in motion: Mg, Cu and Ta

Temperature effects: Mg, Cu and Ta

Derivation of Taylor-Orowan Equation

Let us consider a crystal slab with dimensions of l_x and l_z in the x and z directions and infinite in y, the dislocation line direction. An upper portion with thickness h, is pushed from left side to the interior by a displacement of b. An edge dislocation is thus created, with the core located at a distance l from the left side.

The shear strain of the slab, γ_{zx}, is non-uniform, but an average over the dislocated volume $l \times 1 \times l_z$ and un-dislocated volume $(l_x - l) \times 1 \times l_z$ can be obtained

$$\gamma_{zx} = \{\frac{b}{l_z} \cdot (l \cdot 1 \cdot l_z) + 0 \cdot [(l_x - l) \cdot 1 \cdot l_z]\} / (l_x \cdot 1 \cdot l_z) = \frac{bl}{l_x l_z} = \rho bl$$

The shear strain γ_{zx} is independent of h.

The normal strain ε_x, also by average, takes the form

$$\varepsilon_x = \{\frac{b}{l} \cdot (l \cdot 1 \cdot h) + 0 \cdot [l \cdot 1 \cdot (l_z - h)] + [(l_x - l) \cdot 1 \cdot l_z]\} / (l_x \cdot 1 \cdot l_z) = \frac{bh}{l_x l_z} = \rho bh$$

The normal strain ε_x depends on h, not l.

Similarly, if a pre-existing dislocation located at l moves by an arbitrary distance Δl, the shear strain caused is

$$\gamma_{zx} = \rho b(l + \Delta l) - \rho bl = \rho b \Delta l$$

If an edge dislocation moves from left side to right side and exits, the shear strain caused is

$$\gamma_{zx} = \rho b l_x = b / l_z$$

Cu and Ta in pure shear: Dislocation displacement-strain curve

The dislocation motion in Cu and Ta in pure shear are similar to that in Mg, as shown in Figs. S1.

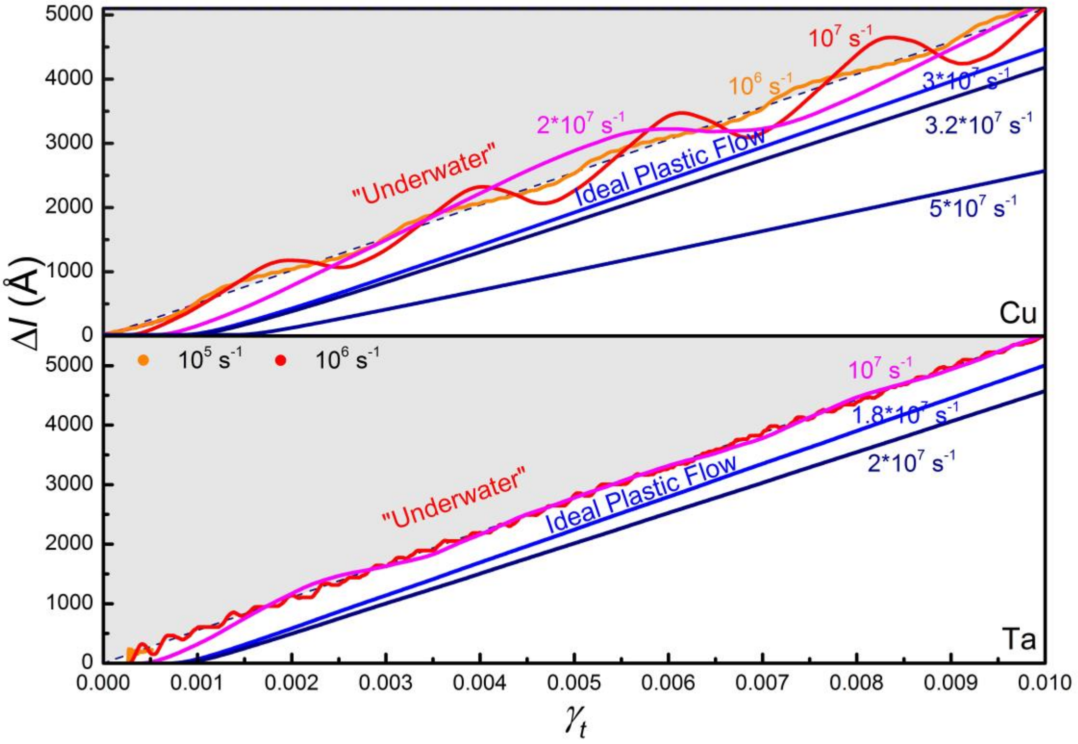

Fig. S1. Dislocation displacement ~ strain (Δl ~ γ_t) curve for Cu and Ta crystals containing a single edge dislocation sheared at various strain rates. Overshooting of the equilibrium position (diagonal) due to dislocation inertia causes negative stress. The overshot region (“Under Water”) is dark-greyed.

Sample size effects: Mg

In order to evaluate sample size effects, the sample length (gliding direction) is doubled in Mg. Constant strain rate shear and a combined loading condition of constant stress rate (10^{-6} G ps^{-1}), constant stress (10^{-3} G) and stress relaxation are performed for an edge dislocation using the Sun EAM [1] potential. Similar results are obtained with the same conclusions drawn, as shown in Figs. S2 and S3.

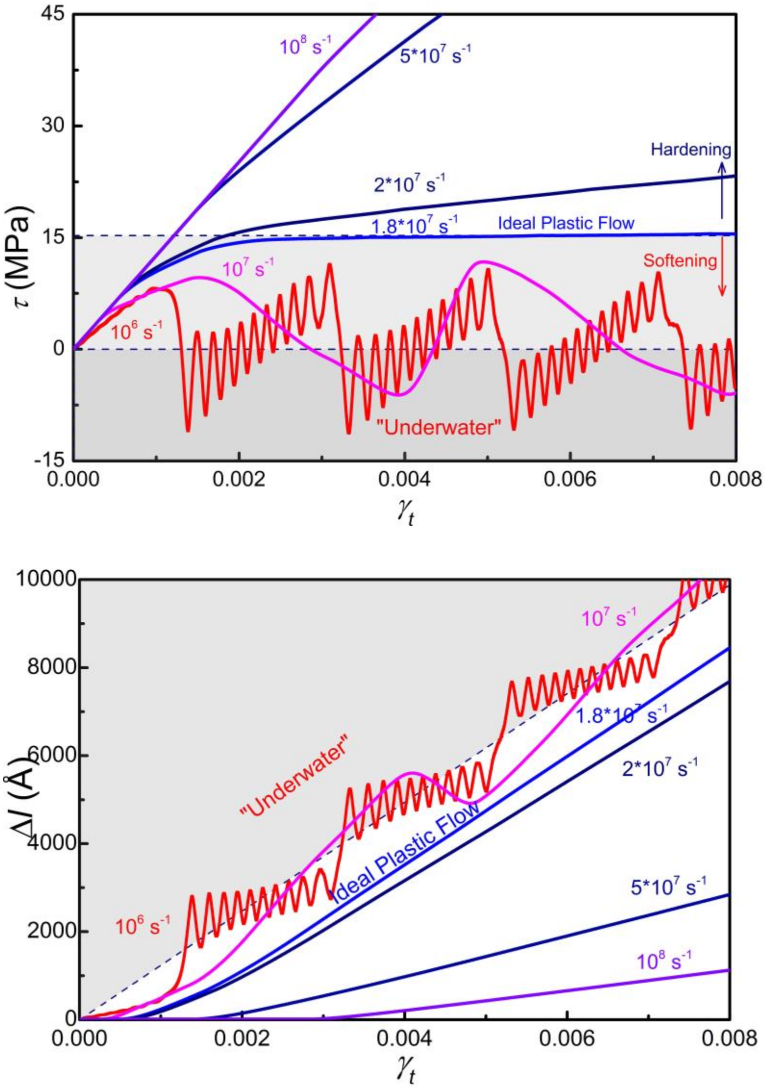

Fig. S2. Stress ~ strain (τ ~ γ_t) responses and dislocation displacement ~ strain (Δl ~ γ_t) curves for larger Mg crystal containing a single edge dislocation sheared at various strain rates. Softening region is light-greyed and negative stress region ("Under Water") is dark-greyed. In all cases, shear stress oscillates around the zero-stress axis in the softening region due to inertia. In the Δl ~ γ_t plot, dislocation overshooting the equilibrium position (diagonal) due to dislocation inertia is seen. The overshot region ("Under Water") is also dark-greyed.

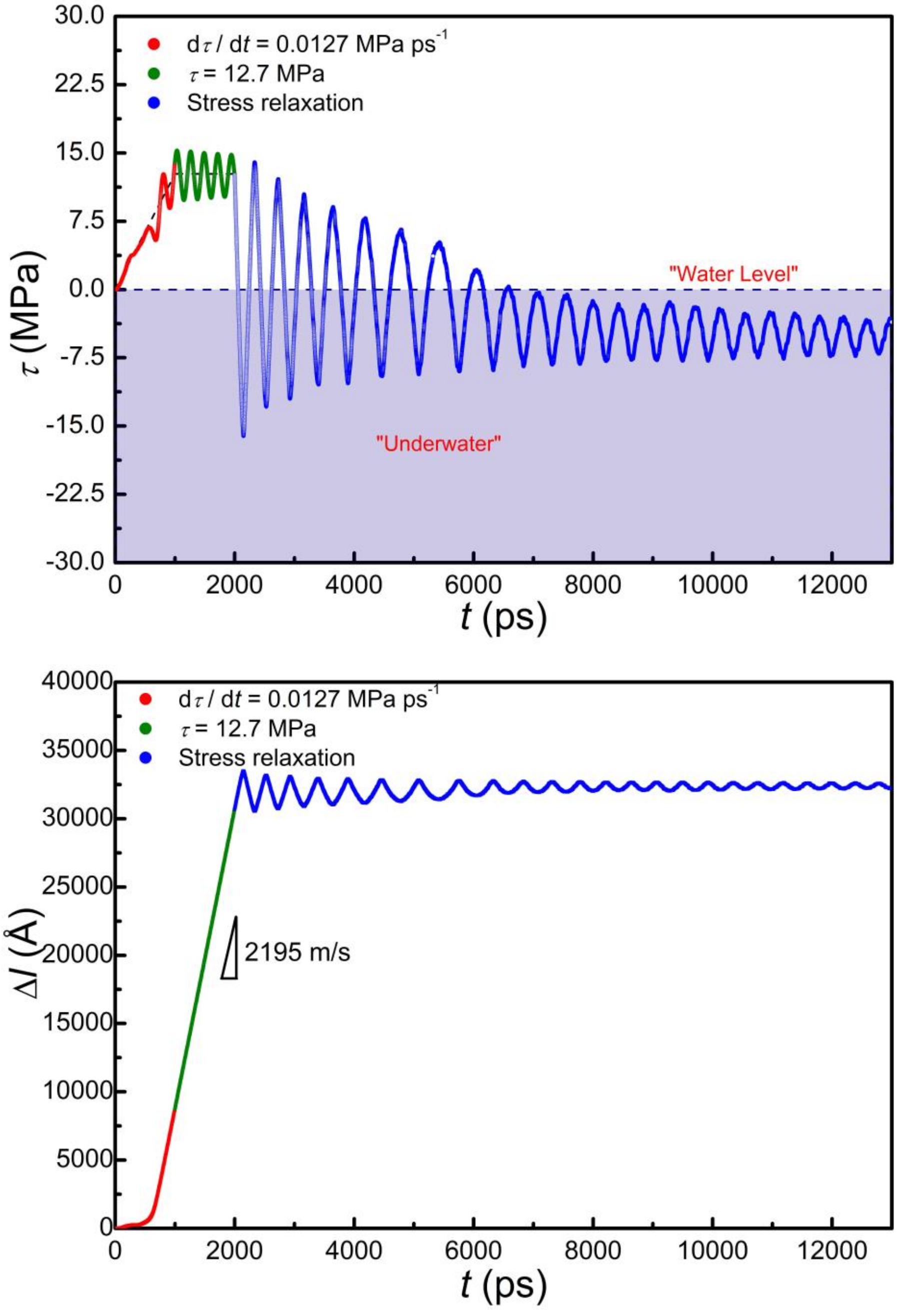

Fig. S3. Stress evolution ($\tau \sim t$) and dislocation displacement ~ time ($\Delta l \sim t$) curves for larger Mg crystal containing a single edge dislocation in a combined shear loading condition of constant stress rate (10^{-6} G ps^{-1}), constant stress (10^{-3} G) and stress relaxation. Negative stress region ("Under Water") is dark-greyed. The shear stress oscillates around the zero-stress axis at a frequency of gigahertz during stress relaxation due to inertial motion of the dislocation. The steady velocity obtained, 2195 m/s, is very close to that for a smaller sample, 2205 m/s.

Potential effects: Mg

The adoption of potential is crucial for Mg in some cases. In order to evaluate the potential effects, the MEAM potential for Mg [2] is also adopted. Constant strain rate loading and a combined loading condition of constant stress rate (10^{-6} G ps^{-1}), constant stress (10^{-3} G) and stress relaxation are also performed for an edge dislocation. Similar results are obtained for all cases, with the same conclusions drawn, as shown in Figs. S4 and S5.

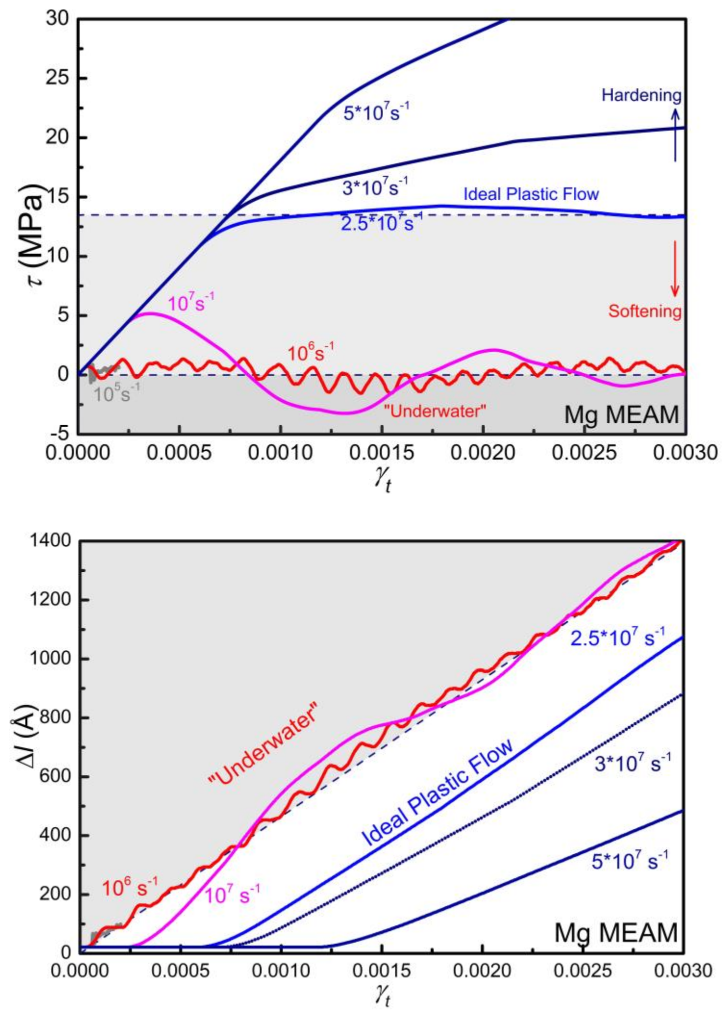

Fig. S4. Stress ~ strain ($\tau \sim \gamma_t$) responses and dislocation displacement-strain ($\Delta l \sim \gamma_t$) curves for Mg crystal containing a single edge dislocation sheared at various strain rates, using the MEAM potential. Softening region is light-greyed and negative stress region ("Under Water") is dark-greyed. Shear stress oscillates around the zero-stress axis in the softening region due to inertia. In the $\Delta l \sim \gamma_t$ plot, dislocation overshooting the equilibrium position (diagonal) due to dislocation inertia is seen. The overshot region ("Under Water") is also dark-greyed.

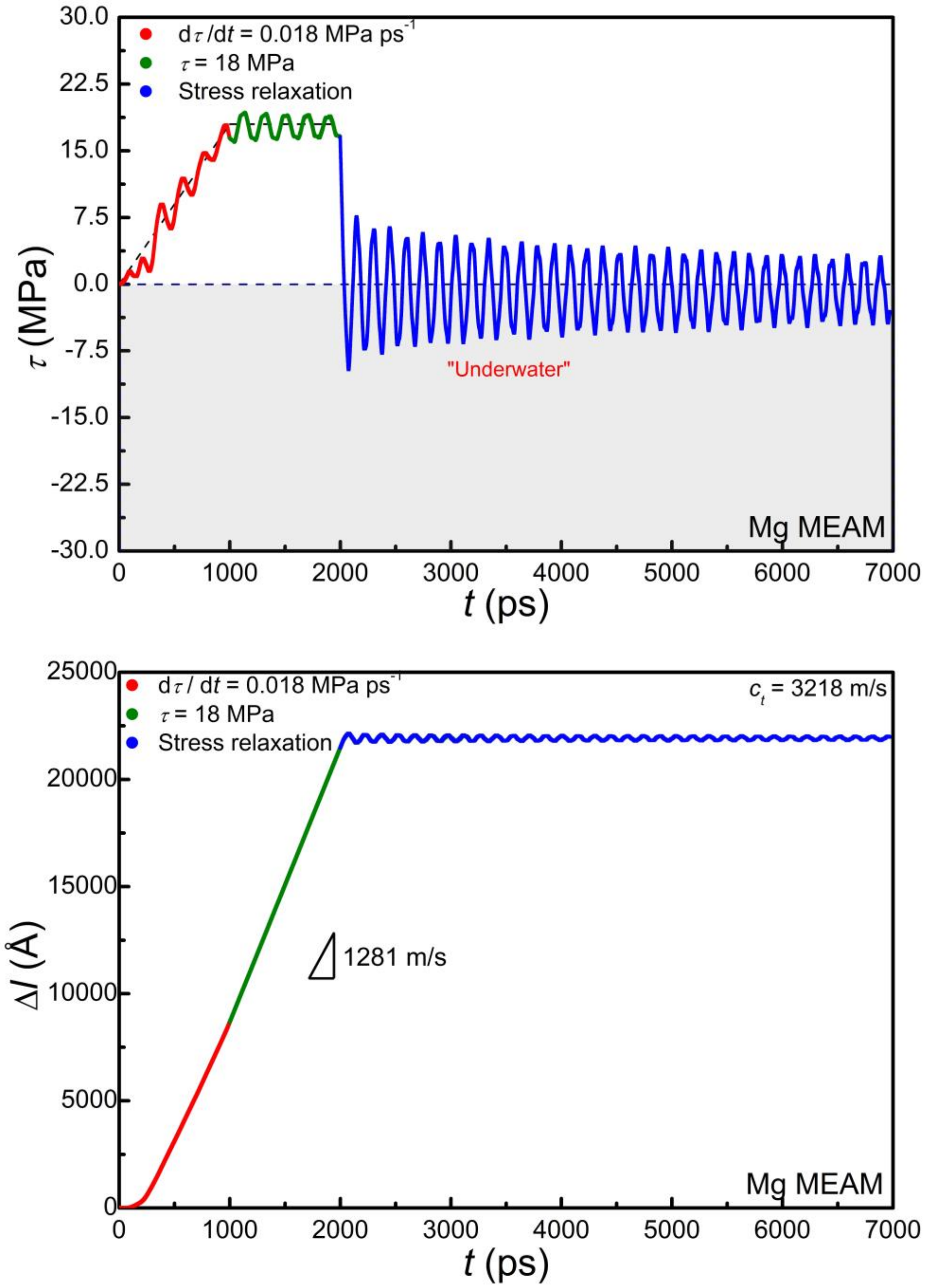

Fig. S5. Stress evolution ($\tau \sim t$) and dislocation displacement ~ time ($\Delta l \sim t$) curves for Mg crystal containing a single edge dislocation in a combined loading condition of constant stress rate (10^{-6} G ps^{-1}), constant stress (10^{-3} G) and stress relaxation, using the MEAM potential. Negative stress region ("Under Water") is dark-greyed. The shear stress oscillates around the zero-stress axis at a frequency of gigahertz during stress relaxation due to inertial motion of the dislocation. The steady dislocation velocity obtained, 1281 m/s, is about only half of that predicated by the EAM potential [1].

Energies of dislocation at rest and in motion: Mg, Cu and Ta

The spatial distributions of the potential energy, kinetic energy and particle velocities of the atoms in the glide plane of dislocations in Mg, Cu and Ta are shown in Figs. 6 and 7. It is seen that, there is always considerable kinetic energy stored in the moving core of a dislocation.

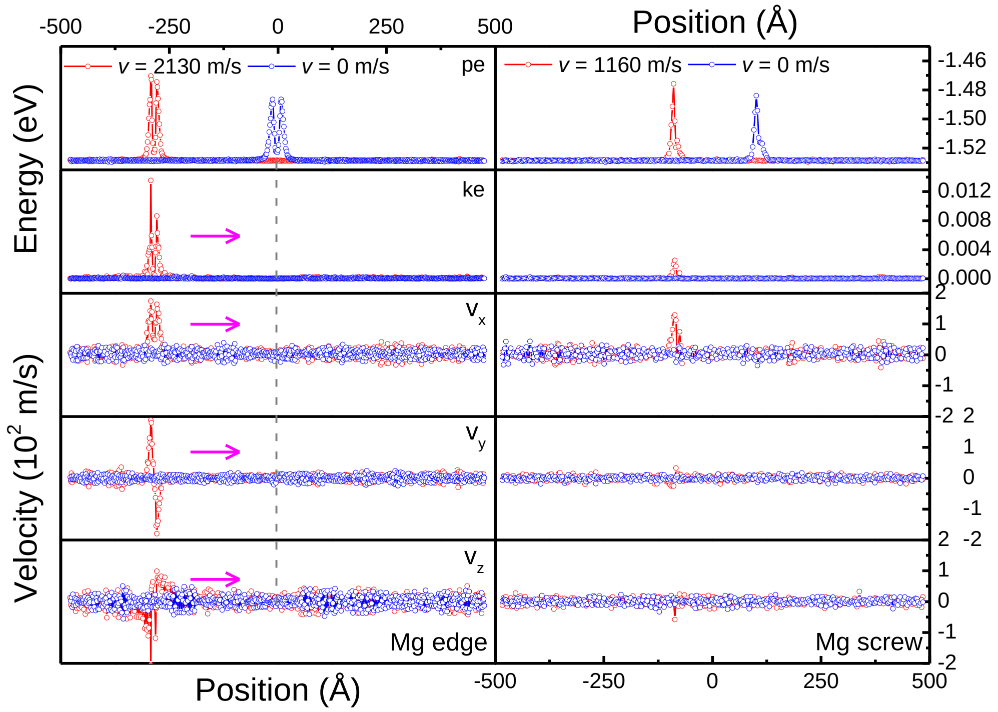

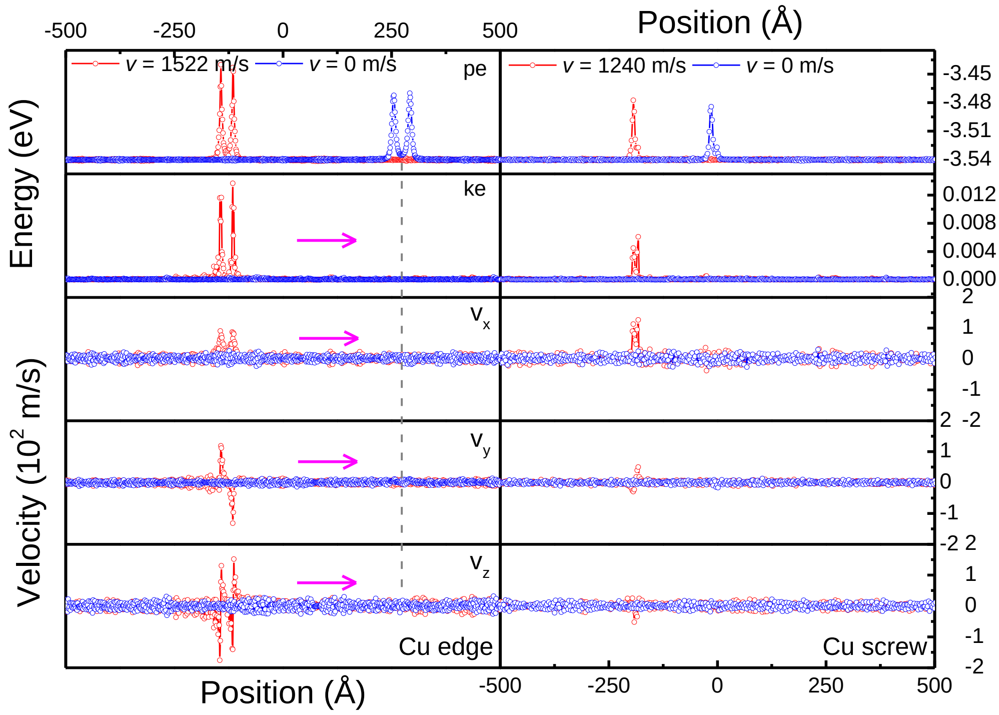

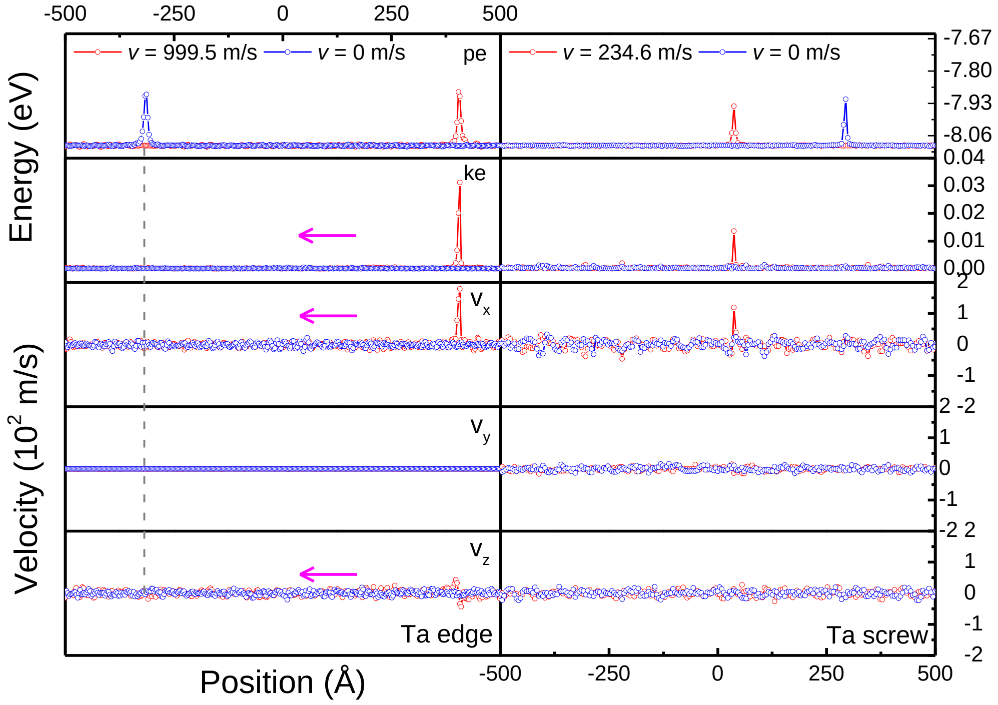

Fig. S6. Spatial distributions of the potential energy *pe*, kinetic energy *ke* and particle velocities *Vx*, *Vy* and *Vz* of the atoms in the glide plane of an edge and a screw dislocation at different velocities in Mg, Cu and Ta. Considerable kinetic energy is carried in the core of the moving dislocation in all cases.

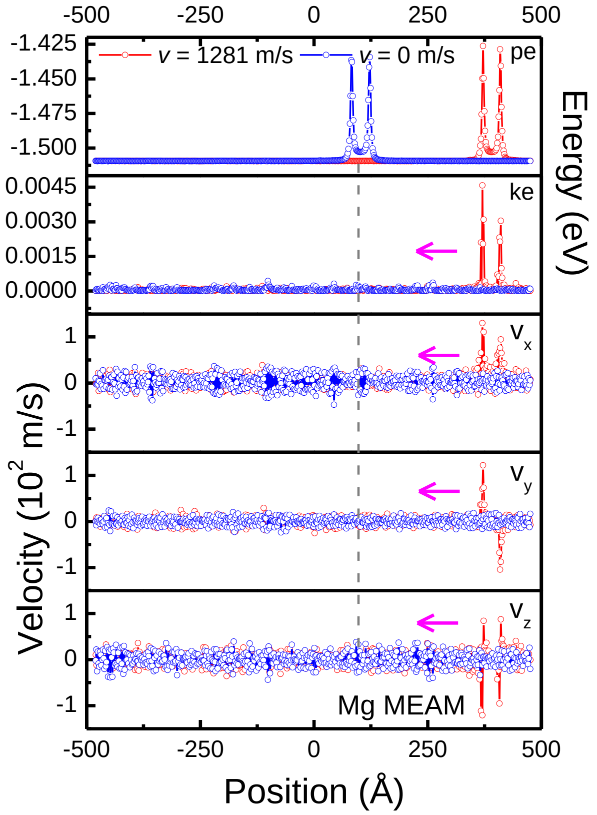

Fig. S7. Spatial distributions of the potential energy *pe*, kinetic energy *ke* and particle velocities *Vx*, *Vy* and *Vz* of the atoms in the glide plane of an edge dislocation at different velocities in Mg, using the MEAM potential. Considerable kinetic energy is carried in the core of the moving dislocation.

Temperature effects: Mg, Cu and Ta

In order to evaluate the temperature effects, a higher temperature, 77K is adopted for an edge dislocation in Mg, Cu and Ta. As temperature increases, rapidly increased phonon density results in enhanced phonon drag; hence inertia effect, though still exists, becomes less significant and sometimes stays hidden from dissipative motion.

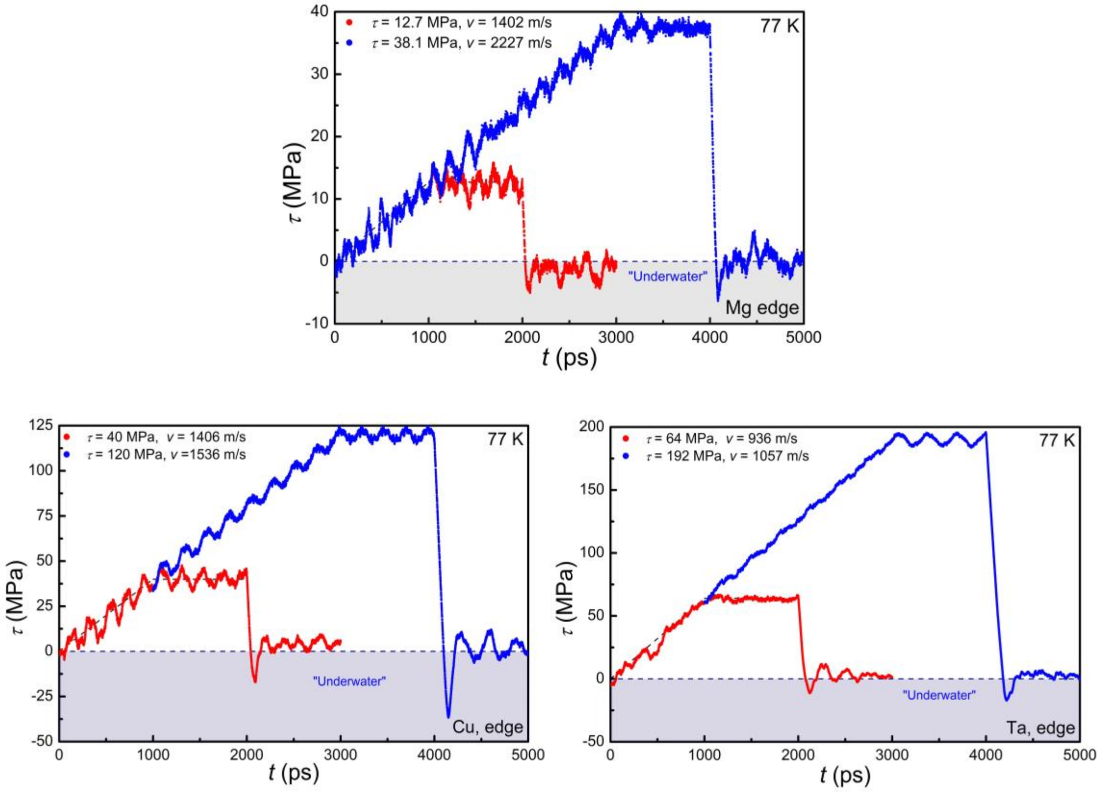

Fig. S8. Stress evolution ($\tau \sim t$) for Mg, Cu and Ta crystals containing an edge dislocation in a combined loading condition of constant stress rate (10^{-6} G ps^{-1}), constant stress (10^{-3} G , red curve; 3×10^{-3} G , blue curve) and stress relaxation at 77K. The steady, also maximum dislocation velocity is also given. Significant increase in phonon drag is seen only in Mg (maximum velocity drops from 2205 m/s at 0K to1402 m/s at 77K), due to its high MSR velocity, 162 m/s at 77K. In all cases negative shear stress is indeed observed during stress relaxation due to inertial motion of the dislocation, but with less significance than that at 0 K.

References:

1. Sun, D. Y., Mendelev, M.I., Becker, C.A., Kudin, K., Haxhimali, T., Asta, M., Hoyt, J.J., Karma, A. & Srolovitz, D.J. Crystal-melt interfacial free energies in hcp metals: A molecular dynamics study of Mg. *Phys. Rev B* **73,** 024116 (2006).
2. Wu, Z., Francis, M. F. & Curtin, W. A. Magnesium interatomic potential for simulating plasticity and fracture phenomena. *Model. Simul. Mater. Sci. Eng.* **23,** 015004 (2015).
